# Purinergic Receptors Crosstalk with CCR5 to Amplify Ca^2+^ Signaling

**DOI:** 10.1007/s10571-020-01002-1

**Published:** 2020-11-20

**Authors:** Mizuho Horioka, Emilie Ceraudo, Emily Lorenzen, Thomas P. Sakmar, Thomas Huber

**Affiliations:** 1 Tri-Institutional Program in Chemical Biology, New York, NY 10065 USA; 2 Laboratory of Chemical Biology and Signal Transduction, The Rockefeller University, 1230 York Ave., New York, NY USA

**Keywords:** GPCR signaling, CCR5, Chemokines, Purinergic receptors, G protein bias, Crosstalk

## Abstract

Many G protein-coupled receptors (GPCRs) signal through more than one subtype of heterotrimeric G proteins. For example, the C–C chemokine receptor type 5 (CCR5), which serves as a co-receptor to facilitate cellular entry of human immunodeficiency virus 1 (HIV-1), normally signals through the heterotrimeric G protein, Gi. However, CCR5 also exhibits G protein signaling bias and certain chemokine analogs can cause a switch to Gq pathways to induce Ca^2+^ signaling. We want to understand how much of the Ca^2+^ signaling from Gi-coupled receptors is due to G protein promiscuity and how much is due to transactivation and crosstalk with other receptors. We propose a possible mechanism underlying the apparent switching between different G protein signaling pathways. We show that chemokine-mediated Ca^2+^ flux in HEK293T cells expressing CCR5 can be primed and enhanced by ATP pretreatment. In addition, agonist-dependent lysosomal exocytosis results in the release of ATP to the extracellular milieu, which amplifies cellular signaling networks. ATP is quickly degraded via ADP and AMP to adenosine. ATP, ADP and adenosine activate different cell surface purinergic receptors. Endogenous Gq-coupled purinergic P2Y receptors amplify Ca^2+^ signaling and allow for Gi- and Gq-coupled receptor signaling pathways to converge. Associated secretory release of GPCR ligands, such as chemokines, opioids, and monoamines, should also lead to concomitant release of ATP with a synergistic effect on Ca^2+^ signaling. Our results suggest that crosstalk between ATP-activated purinergic receptors and other Gi-coupled GPCRs is an important cooperative mechanism to amplify the intracellular Ca^2+^ signaling response.

**Electronic supplementary material:**

The online version of this article (10.1007/s10571-020-01002-1) contains supplementary material, which is available to authorized users.

## Introduction

G protein-coupled receptors (GPCRs) are the largest family of transmembrane receptors that mediate many important physiological processes. They are activated by ligand binding on the extracellular side and then couple preferentially to a unique Gα protein sub-family on the intracellular side to activate specific downstream signaling pathways. The Gα subunit in complex with the Gβγ subunits make up the heterotrimeric G proteins. There are four main families of Gα proteins: Gi/o, Gq/11, Gs, and G12/13, which we refer to as Gi, Gq, Gs, and G12. Once activated by the GPCR, the G protein dissociates into the Gα and Gβγ subunits, and both of these subunits can go on to mediate downstream effector activity by activating or inhibiting enzymes or channels. Some GPCRs do not couple to a unique Gα protein and have the ability to signal through more than one Gα protein class (Asano et al. 1984). GPCR signaling is further complicated by the ability for receptor crosstalk, which can modify downstream function (Werry et al. 2003). Some GPCRs also exhibit ligand-dependent G protein-subtype bias where the nature of the agonist dictates G protein-subtype coupling in the same cellular background (Lorenzen et al. 2018).

The C–C chemokine receptor type 5 (CCR5) is one such example of a GPCR that displays G protein-subtype signaling bias. The human immunodeficiency virus 1 (HIV-1) uses CCR5 as a co-receptor to infect immune cells (Barmania and Pepper 2013). The native chemokine ligand, regulated on activation, normal T cell expressed and secreted (RANTES) has the ability to inhibit HIV-1 entry, but has very low potencies and low affinity for CCR5 (Raport et al. 1996). A synthetic analog of RANTES, chemically modified at the N-terminal tail called PSC-RANTES, has increased potency for blocking HIV-1 entry (Hartley et al. 2004). PSC-RANTES shows picomolar potencies and is a "super" agonist for CCR5 (Gaertner et al. 2008). PSC-RANTES is more efficacious than RANTES at increasing intracellular Ca^2+^ and ultimately causing internalization of CCR5 (Hartley et al. 2004). CCR5 canonically couples to and activates Gi, which are primarily responsible for adenylyl cyclase inhibition. However, the liberated Gβγ subunits from activated Gi are also capable of stimulating Ca^2+^ flux, albeit less effectively, so it is not clear if the Ca^2+^ flux is a result of Gq activation or from the release of Gβγ subunits following Gi activation (Flanagan 2014). Previously, CCR5 was shown to be able to switch between Gi and Gq signaling (Molon et al. 2005). Furthermore, RANTES and RANTES analogs were shown to activate different Gα families in the same human cell line (Lorenzen et al. 2018). All analogs could induce Gi activation, but only some (namely PSC-RANTES and 6P4-RANTES) could activate Gq proteins.

There have been many examples of crosstalk between Gi- and Gq-coupled receptors that modify Ca^2+^ signaling in cells, ranging from Gi-coupled δ-opioid receptors to chemokine receptors. For example, DPDPE activation of δ-opioid receptors and DAMGO activation of μ-opioid receptors did not alter free intercellular Ca^2+^ concentrations unless Gq-coupled M3 muscarinic receptors were first activated by carbachol or oxotremorine-M (Connor and Henderson 1996; Yeo et al. 2001). Similarly, leucine enkephalin could only induce a Ca^2+^ flux through δ-opioid receptors when ATP had pre-stimulated the phospholipase C (PLC)/Ca^2+^ system via P2 purinergic receptors (Okajima et al. 1993). Interestingly, purinergic receptors also crosstalk with the chemokine receptors. Studies have reported that cells expressing chemokine receptors such as C-X-C motif chemokine receptor 2 (CXCR2) and C–C chemokine receptor type 4 (CCR4) have seen enhanced release of intracellular Ca^2+^ with ATP pre-stimulation, which activates endogenous purinergic receptors (Corriden and Insel 2010; Rosethorne et al. 2004; Sivaramakrishnan et al. 2012; Werry et al. 2002).

Here, we show that the Ca^2+^ flux resulting from CCR5 stimulation by the Gi-biased native chemokine RANTES, and the Gq-biased synthetic super agonist, PSC-RANTES, are both amplified by crosstalk with endogenous purinergic receptors. Purinergic receptors bind to either ATP (P2 P-type receptors) or its breakdown product, adenosine (P1 A-type receptors). The P2Y family of purinergic receptors are GPCRs that couple to specific G proteins (Burnstock 2006). As hypothesized, ATP priming of HEK293T cells expressing CCR5 enhanced both RANTES and PSC-RANTES-induced Ca^2+^ flux, although a larger enhancement was seen for RANTES. This enhancement by ATP pre-stimulation was decreased or abolished when cells were incubated with compounds that blocked endogenous purinergic receptors. The decrease in enhancement was much more dramatic for RANTES-stimulated Ca^2+^ flux. The larger effect is no doubt due to RANTES coupling to Gi, which leads to less effective Ca^2+^ signaling, and so the enhancement through crosstalk to other purinergic receptors is more pronounced. Furthermore, we noted that the effect was especially pronounced for the inhibitor NF157, which blocks the P2Y receptor, P2Y_11_. Complementing the observations of others, we have shown that ATP priming activates P2Y receptors and the crosstalk between these Gq-coupled receptors and canonically Gi-coupled CCR5 enhances Ca^2+^ signaling in cells. Agonist-dependent exocytotic release of lysosomal contents results in the simultaneous release of ATP, which activates purinergic receptors (Cekic and Linden 2016; Dosch et al. 2018; Ferrari et al. 2016; Reddy et al. 2001). Furthermore, the secretory release of GPCR ligands, such as chemokines and opioids, also benefit from the release of ATP from secretory vesicles (Kronlage et al. 2010; Samie and Xu 2014). Having two simultaneous local diffusible signals leads to a strong local activation of Ca^2+^ signaling in target cells and a sharpened response. In both cases, the crosstalk between ATP-activated purinergic receptors and other Gi-coupled GPCRs acts as a cooperative step to amplify the intracellular Ca^2+^ signaling response.

## Materials and Methods

### Materials

NF157, MRS2500, suramin, AR-C 118925XX, CGS15943, pertussis toxin (PTX), ATP, and ATPγS were from Tocris Bioscience (Bristol, UK). YM-254890 (YM) was from Wako Pure Chemical Industries (Richmond, VA). Bovine serum albumin (BSA) fraction V fatty acid-free was from Roche (Basel, Switzerland). Poly-d-lysine and apyrase were from Sigma-Aldrich (St. Louis, MO). Carbachol was from Abcam (Cambridge, UK). Triton X-100 was from Polysciences (Warrington, PA). RANTES was from Peprotech (Rocky Hill, NJ) and RANTES analog, PSC-RANTES was a gift from Oliver Hartley (Université de Genève). HEK293T cells were from American Type Culture Collection (ATCC) (Manassas, VA). Dulbecco’s Modified Eagle’s Medium GlutaMAX (DMEM), FluoroBrite DMEM, Dulbecco’s phosphate-buffered saline without Ca^2+^ and Mg^2+^ (DPBS), Hanks' Balanced Salt solution (HBSS), and HEPES buffer were from Fisher Scientific (Hampton, NH). Lipofectamine 2000 and trypsin–EDTA (0.25%, phenol red) were from ThermoFisher Scientific (Waltham, MA). Fetal bovine serum (FBS) was from Gemini Bio-Products (West Sacramento, CA). Clear and clear-bottom black 384-well microplates were from Greiner (Monroe, NC). FLIPR Calcium 6 Assay and Flexstation II 384 Plate Reader were from Molecular Devices (San Jose, CA). 384-well transfer tips for the Flexstation were from Axygen (Union City, CA).

### Cell Culture and Transfections

HEK293T cells were maintained in DMEM GlutaMAX supplemented with 10% FBS (passage numbers 5 to 16) at 37 °C under 5% CO_2_. Cells were transiently transfected with a synthetic vector encoding human CCR5 cDNA in pcDNA3.1(+) with a C-terminal 1D4 epitope tag (TETSQVAPA). Transfections were done directly ‘in-plate’ in 384-well microplates using Lipofectamine 2000 according to manufacturer’s instructions with some modifications. Briefly, 30 ng per well of CCR5-1D4 plasmid DNA was mixed in DMEM GlutaMAX (no FBS). In a separate mixture, the total Lipofectamine 2000 (2.5 µL per µg DNA) was mixed in DMEM GlutaMAX (no FBS) and incubated for 5 min. The Lipofectamine 2000/DMEM mixture was mixed with the DNA/DMEM and incubated for 20 min. Cells were then trypsinized, re-suspended in DMEM supplemented with 20% FBS, and counted. Cells were mixed with the DNA/Lipofectamine 2000/DMEM mixture, and directly plated onto 0.01% poly-d-lysine coated, black, clear-bottom, tissue culture treated 384-well plates at a density of 30,000 cells per well in 20 µL total volume.

### Ca^2+^ Measurements Using FLIPR Calcium 6 Dye

HEK293T cells were transfected as described above and incubated at 37 °C under 5% CO_2_ for 24 h. On the day of the experiment, FLIPR Calcium 6 dye was diluted to a 4 × concentration in Hanks' Balanced Salt solution supplemented with 20 mM HEPES, pH 7.4 (HBSS-H) with 0.4% BSA. Then, 10 µL of the 4 × FLIPR Calcium 6 dye was added to each of the wells. Further, 10 µL of YM at a final concentration of 1 µM in HBSS-H with 0.4% BSA was added to the appropriate wells. The plate was placed back into the incubator at 37 °C under 5% CO_2_ for 1.5 h. In the meantime, the ligand plate was prepared in a clear 384-well microwell plate, with 5 × concentration of each ligand in HBSS-H with 0.4% BSA. The FlexStation II 384 Plate Reader (Molecular Devices) will take out 10 µL from these wells and inject into the appropriate wells in the assay plate with the cells. The ligands used were PSC-RANTES (100 nM final), RANTES (100 nM final), ATP (10 µM final), carbachol (1 mM final), ATPγS (10 µM final) as well as HBSS-H + 0.4% BSA (buffer only). After 1.5 h incubation, 10 µL of purinergic receptor inhibitors, NF157, MRS2500, suramin, and AR-C 118925XX at a final concentration of 100 µM in HBSS-H with 0.4% BSA were added to the appropriate wells. Note that 10 µL of apyrase at a final concentration of 0.2 U/well in HBSS-H with 0.4% BSA were added to appropriate wells as well. For single injection experiments, additional inhibitors PTX (100 ng/mL final, 16 h) and CGS15943 (100 µM final, 30 min) were also incubated.

**Fig. 1:**
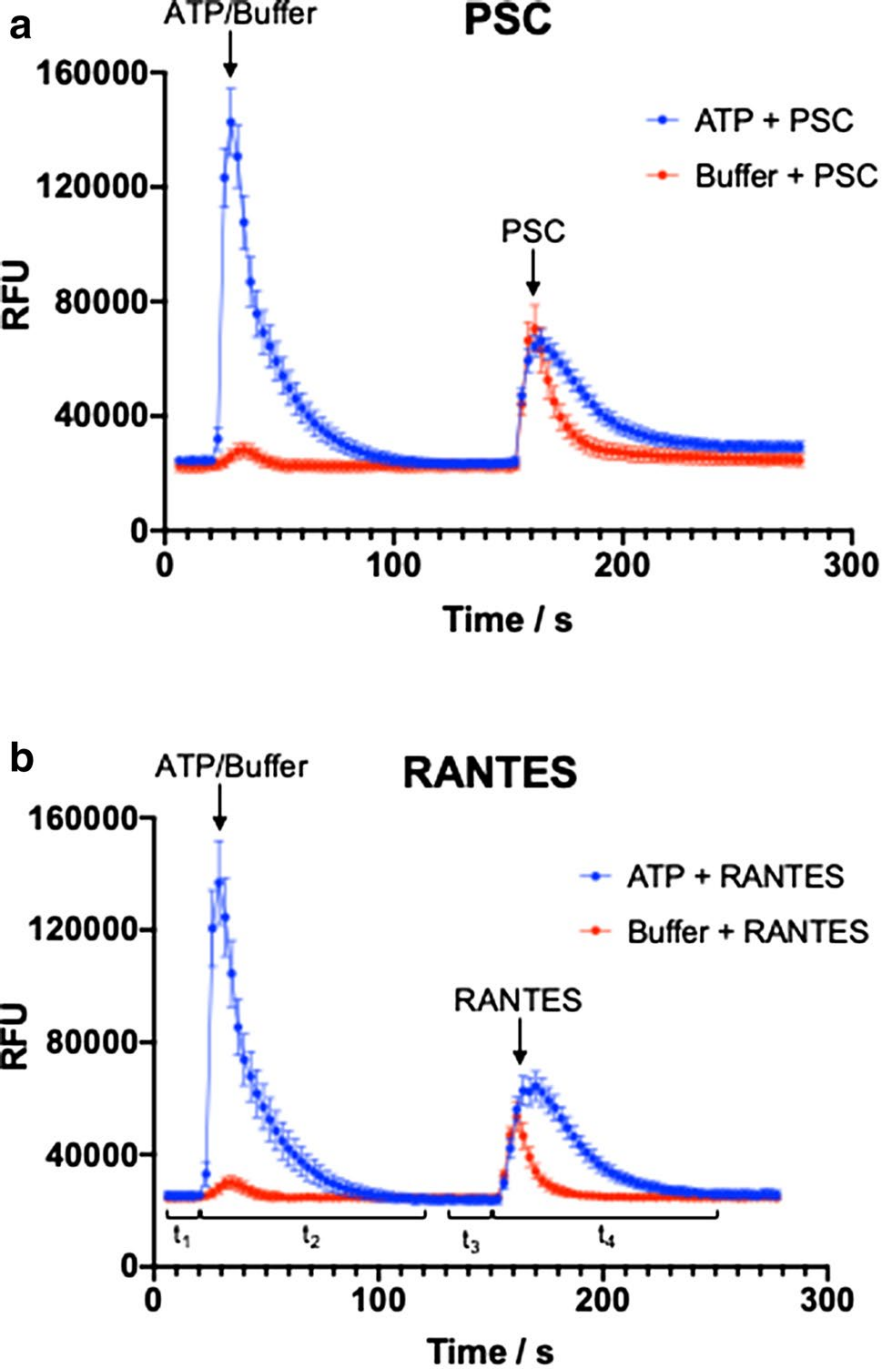
ATP pre-stimulation causes detectable changes in Ca^2+^ flux of CCR5-expressing cells stimulated by PSC-RANTES and RANTES. CCR5-encoding HEK293T cells were first injected with 10 µM of ATP (blue) or buffer (red) at time 17 s, indicated with the first arrow. ATP injection causes a sharp increase in fluorescence, while buffer injection causes a negligible flux. Then, at time 150 s, cells were subjected to a second injection of agonist, indicated with the second arrow. With the ATP pre-stimulation, stimulation by both PSC-RANTES (PSC, top) and RANTES (bottom) shows a larger and sustained fluorescence or Ca^2+^ flux. The increase in intracellular Ca^2+^ levels for injection 1 was calculated as the mean RFU between 20 and 120 s (*t*_2_, raw injection signal) minus mean RFU between 0 and 20 s (*t*_1_, basal signal), *t*_2_ − *t*_1_. Similarly, injection 2 was calculated as the mean RFU between 150 and 250 s (*t*_4_) minus mean RFU between 130 and 150 s (*t*_3_), *t*_4_ − *t*_3_. Data are mean ± SEM from three independent experiments with four technical replicates each

Prior to measurement, the plate was incubated at 37 °C for an additional 30 min in a pre-warmed FlexStation. Fluorescence readings were collected with excitation at 485 nm and emission at 535 nm. The FlexStation took measurements over a 120 s (single injection experiments) or 250 s (double injection experiments) time course at 2.5 s intervals. For the single injection experiments, 10 µL of the agonists PSC-RANTES, RANTES, ATP, carbachol, ATPγS or buffer were added to the cells at a pipette height of 35 µL at 17 secs. Similarly, for the double injection experiments, at 17 s, the first injection added 10 µL of ATP or buffer to the cells at a pipette height of 20 µL with two trituration steps of 20 µL each. At 150 s, the second injection added 10 µL of agonists to the cells at a pipette height of 20 µL with two trituration steps of 20 µL each. It is important to note that as we have two compound transfer steps, half of the plate was read at a time to account for the number of 384-well transfer tips per rack. This was also to ensure the cells were not incubating in the FLIPR Calcium 6 dye for over 2 h as this may lead to cytotoxicity. Relative fluorescence units (RFU) are calculated for injection 1 as the mean signal between 20 and 120 s (*t*_2_, raw injection signal) minus mean signal between 0 and 20 s (*t*_1_, basal signal), and for injection 2 as the mean signal between 150 and 250 s (*t*_4_, raw injection signal) minus mean signal between 130 and 150 s (*t*_3_, basal signal) (Fig. 1).

### Ca^2+^ Measurements Using GCaMP6s

GCaMP6s is a genetically encoded Ca^2+^ indicator composed of a circularly permuted green fluorescent protein (cpGFP), calmodulin and the M13 domain of the myosin light chain kinase (Chen et al. 2013). Calmodulin binds up to four Ca^2+^ ions and induces a conformational change to increase GFP fluorescence, which can be conveniently monitored at the same wavelengths as those of the FLIPR Calcium 6 dye. HEK293T cells were transfected as described above, transfecting 20 ng per well of CCR5-1D4 plasmid DNA and 10 ng per well of GCaMP6s plasmid DNA. DMEM media without phenol red, called FluoroBrite DMEM (supplemented with 30 mM HEPES), was used for these transfections as the phenol red can interfere with GFP fluorescence signals. Cells were incubated at 37 °C under 5% CO_2_ for 24 h. On the day of the experiment, the media from half of the wells were aspirated carefully using the 8-port manifold. To those wells, 30 µL of HBSS-H with 0.4% BSA was added and to the other half 10 µL of HBSS-H with 0.4% BSA was added. Further, 10 µL of YM at a final concentration of 1 µM in HBSS-H with 0.4% BSA was added to the appropriate wells. The plate was placed back into the incubator at 37 °C under 5% CO_2_ for 1.5 h. In the meantime, the ligand plate was prepared as described above. After 1.5 h incubation, the purinergic receptor inhibitors were added to the appropriate wells. For single injection experiments, CGS15943 was added at three concentrations (1 µM, 10 µM, and 100 µM final) for 30 min.

Prior to measurement, the plate was incubated at 37 °C for an additional 30 min in a pre-warmed FlexStation II 384 Plate Reader. Fluorescence readings were collected at 485 nm excitation and 535 nm emission using the same settings to stimulate the cells and collect the data as outlined above. The same analysis as above was conducted to obtain the corrected injection values.

### Total Cell Count by Triton X-100 Lysis

HEK293T cells transfected with 20 ng per well of CCR5-1D4 plasmid DNA and 10 ng per well of GCaMP6s plasmid DNA were incubated at 37 °C under 5% CO_2_ for 24 h in FluoroBrite DMEM, as described above. On the day of the experiment, the steps described above were followed to prepare the assay plate as well as the ligand plate. In the ligand plate, the CCR5 ligands were prepared along with 0.1% Triton X-100 (final). The FlexStation added 10 µL of ligand to the cells at 17 s (first injection) and then 10 µL of Triton X-100 at 150 s (second injection). Similar analyses were conducted to obtain corrected mean RFU values. Injection 1 is calculated as the mean signal between 20 and 120 s (*t*_2_, raw injection signal) minus mean signal between 0 and 20 s (*t*_1_, basal signal), as above. However, for injection 2 the mean signal between 0 and 20 s (*t*_1_, basal signal) is subtracted from the mean signal between 150 and 250 s (*t*_4_, raw injection signal) as we wish to calculate the total increase in fluorescence to quantify total cell count in each well.

### Data Analysis

Data analysis was performed using SoftMax Pro Software version 5, Microsoft Excel, and GraphPad Prism version 8.

## Results

### ATP Pre-stimulation Causes Detectable Changes in Ca^2+^ Flux of CCR5-Expressing Cells Stimulated by PSC-RANTES and RANTES

First, we wanted to assess if we could detect the effect of ATP pre-stimulation on the increase in intracellular Ca^2+^ levels induced by stimulation by a second ligand. HEK293T cells were transiently transfected with CCR5 and intracellular Ca^2+^ levels were detected by FLIPR Calcium 6 assay dye, a permeable dye sensitive to Ca^2+^. The cells were first subject to an injection of either 10 µM of ATP or buffer at time 17 s. Then, 130 s after this first injection, cells were subject to a second injection of either PSC-RANTES (100 nM final), RANTES (100 nM final), ATP (10 µM final), carbachol (1 mM final), ATPγS (10 µM final) or buffer only, and the increase in intracellular Ca^2+^ levels were monitored. The concentrations of these agonists cause maximal Ca^2+^ flux. In particular, the EC_50_ values for PSC-RANTES (25 nM) and RANTES (32 nM) were previously determined by fitting a dose–response curve for CCR5-mediated Ca^2+^ flux stimulated by these two agonists (Lorenzen et al. 2018). Figure 1 shows sample raw data, with increases in fluorescence upon the two injections. First, we notice that the initial ATP injection gives a pronounced and rapid increase in fluorescence, consistent with the purinergic receptors being activated and causing a Ca^2+^ flux. On the other hand, the control buffer injection causes a small, negligible flux. This may be an artifact of the injection and mixing, which may release adenine nucleotides from distal pools and cause a slight flux.

Upon injection with the chemokines RANTES and PSC-RANTES, there is another increase in fluorescence, consistent with the CCR5 receptors being activated and causing a Ca^2+^ flux. These chemokines cause a flux if injected on their own, without an ATP pre-stimulation, but with the pre-stimulation, there is a larger and more sustained fluorescence signal, as seen by the difference in shape of the peaks. For PSC-RANTES, the initial increase in fluorescence, as in the height of the peak, are the same for both ATP and buffer pre-stimulated cells. For RANTES, however, the ATP pre-stimulation causes an increase in the amplitude of the fluorescence peak. However, what is striking is that the area under the curve is much larger for both peaks with the ATP pre-stimulation. Without ATP-pre-stimulation, the fluorescence signal decays rapidly, whereas with the pre-stimulation there seems to be a more sustained signaling and a slower decay.

### ATP Pre-stimulation of CCR5-Expressing Cells Increases PSC-RANTES and RANTES-Induced Ca^2+^ Flux, Which is Significantly Decreased by Incubation with Purinergic Receptor Inhibitors

HEK293T cells transiently transfected with CCR5 were treated with a variety of purinergic receptor inhibitors (Table 1). Purinergic receptors bind to either ATP (P2 P-type receptors) or its breakdown product, adenosine (P1 A-type receptors). For our experiments, we used inhibitors that are specific for the P2Y subtypes of purinergic receptors, as well as more general non-selective inhibitors. MRS2500 is a highly selective inhibitor of the P2Y_1_ receptor, AR-C 118925XX is a selective inhibitor of the P2Y_2_ receptor, and NF157 is a potent inhibitor of the P2Y_11_ receptor that also shows some activity toward the P2X_1_ receptor. These receptors are clustered in sequence homology and preferentially couple to Gq, although P2Y_11_ has been shown to couple to Gs as well (Costanzi et al. 2004). The P2Y_1_ receptor is stimulated by ADP, and ATP to a lesser extent, while P2Y_2_ is stimulated by both ATP and UTP, and P2Y_11_ is only stimulated by ATP (Jacobson et al. 2009). Suramin is a non-selective P2 purinergic inhibitor, apyrase is a tri and diphosphohydrolase that hydrolyses ATP and ADP into AMP, and YM-254890 (YM) is a Gq selective inhibitor. Although it has been suggested that YM, along with a similar Gq selective inhibitor, UBO-QIC/FR900359, inhibits the other G proteins (Gs and Gi), we suspect this may be due to crosstalk of the receptor-activated Gq with the other G proteins (Gao and Jacobson 2016; Peng and Shen 2019). In any case, CCR5-activated Ca^2+^ flux arises predominantly from Gq activation and not from Gβγ subunit release following Gi activation, so any reduction on Ca^2+^ flux seen in our experiments will be due to the YM inhibiting Gq coupling to CCR5, and not Gi coupling (Lorenzen et al. 2018). As the P2Y receptors preferentially couple to Gq as well, YM can decouple their activation from the activation of Gq and inhibit Ca^2+^ flux.

**Table 1:** List of compounds used in this paper with their targets and potencies

Compound	Mechanism	Potency	Cross reactivity
Apyrase	Degradation of ATP and ADP		None
MRS2500	Inhibitor of P2Y_1_ receptor (endogenous ligands ADP ≫ ATP)	IC_50_ = 8.4 ± 0.8 nM (Kim et al. 2003)	None
AR-C 118925XX	Inhibitor of P2Y_2_ receptor (endogenous ligands ATP, UTP)	IC_50_ = 72.1 ± 12.4 nM (Rafehi et al. 2017)	None
NF157	Inhibitor of P2Y_11_ receptor (endogenous ligand ATP)	IC_50_ = 463 ± 59 nM (Ullmann et al. 2005)	P2X_1_ IC_50_ = 63.1 nM
CGS15943	Adenosine receptor antagonist (endogenous ligand adenosine)	IC_50_ = 20 nM at A1, 3 nM at A2 (Williams et al. 1987)	
Suramin	Non-selective antagonist for P2 purinergic receptors	IC_50_ = ~ 1 µM (Dunn and Blakeley 1988)	Also blocks G protein coupling to GPCRs
Pertussis Toxin (PTX)	ADP-ribosylation on α subunits of Gi, Go, and Gt	IC_50_ = 158 ± 40 pg/ml for Gi and 35 ± 8 pg/ml for Go (Liang and Galper 1988)	None
YM-254890 (YM)	Inhibitor of GDP release from the α subunit of Gq	IC_50_ = 0.15 ± 0.04 nM (Nishimura et al. 2010)	

Most are specific P2Y receptor inhibitors, while others are broad-spectrum purinergic receptor antagonists. Apyrase is an ATP-diphosphohydrolase that catalyzes sequential hydrolysis of ATP to ADP and ADP to AMP releasing inorganic phosphate. PTX and YM target G protein coupling to GPCRs. PTX catalyzes ADP-ribosylation on α subunits of G proteins Gi, Go, and Gt, thus preventing them from interacting with receptors. YM on the other hand, inhibits the release of GDP from the α subunit of Gq. The endogenous ligands that activate the purinergic receptors are outlined in parentheses

From the raw data described in Fig. 1, we obtained the corrected mean RFU values for the Ca^2+^ flux induced by the first and second injections. The increase in intracellular Ca^2+^ levels for injection 1 was calculated as the mean RFU between 20 and 120 s (*t*_2_, raw injection signal) minus mean signal between 0 and 20 s (*t*_1_, basal signal), *t*_2_ − * t*_1_. Similarly, injection 2 was calculated as the mean signal between 150 and 250 s (*t*_4_) minus mean signal between 130 and 150 s (*t*_3_), *t*_4_ − *t*_3_. The corrected mean RFU values for the first injection are plotted in Fig. S1. As expected, the buffer injection (black bars) did not give a significant increase in mean RFU. The ATP injection induces Ca^2+^ flux from the CCR5-expressing cells (gray bars), which is decreased by differing amounts upon incubation with the purinergic receptor inhibitors.

**Fig. 2:**
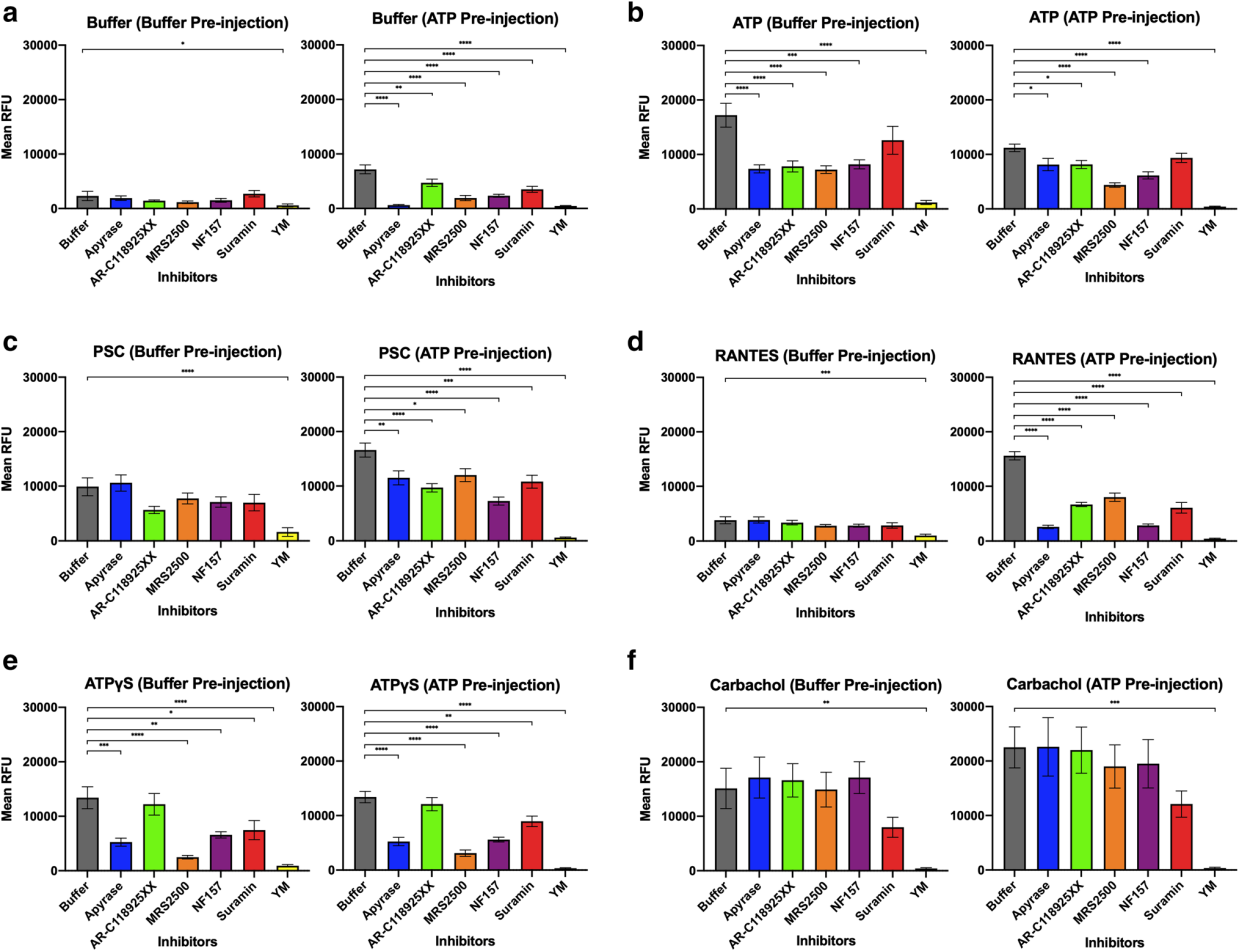
ATP pre-stimulation of CCR5-expressing cells increases PSC-RANTES and RANTES-induced Ca^2+^ flux, which is significantly decreased by incubation with purinergic receptor inhibitors. These graphs show CCR5-encoding HEK293T cells that were pre-injected with buffer (left) or 10 µM ATP (right), followed by a second injection of one of six ligands: **a** buffer, **b** ATP, **c** PSC, **d** RANTES, **e** ATPγS, and **f** carbachol. Prior to injection, cells were incubated with purinergic receptor inhibitors for 30 min (2 h for YM-254890 (YM)), which are listed in the *x*-axis. The increase in intracellular Ca^2+^ levels were monitored as the change in mean RFU and were calculated from the raw data as described in Fig. 1. Each mean RFU was compared to the mean RFU of the buffer incubation case (control, gray). Dunnett’s multiple comparison test was used to assess significance of the ANOVA values and are shown above each bar. Data are mean ± SEM from three independent experiments with four technical replicates each. All inhibitors cause a similar decrease in ATP-induced Ca^2+^ flux as compared to the control cells that were incubated with buffer only (**b**). The notable exception is suramin, the non-selective P2 receptor inhibitor. This decrease is more pronounced in the buffer pre-injected samples as compared to the ATP pre-injected samples. In the buffer pre-injected samples, the inhibitors do not have any significant effect on PSC-RANTES- and RANTES-induced Ca^2+^ fluxes, except for YM (**c**, **d**). ATP pre-stimulation increased the Ca^2+^ flux caused by PSC-RANTES and RANTES, but when cells were incubated with purinergic receptor inhibitors, the PSC-RANTES- and RANTES-induced Ca^2+^ fluxes are decreased significantly, which was not seen without the ATP pre-injection

We then plotted the mean RFU for the second ligand injections (*t*_4_ − *t*_3_), comparing cells pre-stimulated with 10 µM ATP with those that were not (Fig. 2). When CCR5-expressing cells are stimulated with buffer after a buffer pre-injection (Fig. 2a, left) the Ca^2+^ flux is negligible (2320 RFU) and is caused by injection artifacts, as explained above. However, we noticed that after ATP pre-injection, a buffer injection stimulated a significant increase in Ca^2+^ flux (7170 RFU) that is inhibited by incubation with purinergic receptor inhibitors (Fig. 2a, right). A buffer injection should not stimulate the cells, but it seems a second injection with buffer results in signaling only when there is an injection of ATP first. One possible explanation is that the cells deplete the ATP concentration in their proximity due to ATP degrading enzymes. By mixing the media in the wells through a buffer injection, the local ATP concentration is replenished from distal pools, far from the adherent cells. This renewed ATP causes a noticeable Ca^2+^ flux upon buffer injection. This is significantly reduced in the presence of purinergic receptor inhibitors, suggesting that the replenished ATP is activating P2Y receptors to cause Ca^2+^ flux. P2Y receptors couple through Gq, so YM also causes a reduction in Ca^2+^ flux. We were aware that this redistribution effect will be present for all second injections, so we corrected for this effect by treating the second buffer injection as background signal. Thus, the corrected mean RFU for the second buffer injection (*t*_4_ − *t*_3_) was subtracted from the respective second injections of all other ligands incubated with the same inhibitor. This was done for both buffer and ATP pre-injection signals and the results are shown in Fig. S2. The major trends for each ligand are reproducible compared with those pre-correction in Fig. 2, meaning the mixing artifact was not leading to misinformed hypotheses.

In the absence of any inhibitors (buffer incubation) and buffer pre-injection, ATP induces an increase in intracellular Ca^2+^ levels of 17,200 RFU (Fig. 2b, left). This increase in Ca^2+^ flux, however, is reduced following pretreatment with the purinergic receptor inhibitors in a way that mirrors the reduction in Ca^2+^ flux of the first ATP injection (Fig. S1). Apyrase, AR-C 118925XX, MRS2500, and NF157 all decrease ATP-induced Ca^2+^ increase to about half of the Ca^2+^ increase of the untreated cells, to about 8000 RFU. Interestingly, suramin, the non-selective P2 purinergic inhibitor, is an exception and caused an ATP-induced Ca^2+^ increase of 12,600 RFU. ATP pre-stimulation does not increase the Ca^2+^ flux induced by a second ATP injection (Fig. 2b, right). In fact, this is the only reduction in Ca^2+^ flux seen upon ATP pre-stimulation, with a 1.5-fold decrease from 17,200 RFU (buffer pre-stimulation) to 11,200 RFU (ATP pre-stimulation). This is due to desensitization of the receptors and reveals that repeated application of the same ligand will reduce the Ca^2+^ response. Accordingly, the effects of some of the purinergic receptor inhibitors were not as significant as compared to the buffer pre-stimulation case. The Ca^2+^ flux was significantly reduced for cells incubated with NF157 and MRS2500, with mean RFU levels lower than those cells that received a buffer pre-stimulation.

On the other hand, PSC-RANTES, the engineered super agonist of CCR5, induces an increase in intracellular Ca^2+^ levels of 9900 RFU in the absence of inhibitors and without ATP pre-stimulation (Fig. 2c, left). When incubated with the inhibitors, there are slight decreases in the PSC-RANTES-induced Ca^2+^ flux, but none are statistically significant except for cells that were incubated with YM. Interestingly, ATP pre-stimulation increases PSC-RANTES-induced Ca^2+^ flux by about 1.7-fold (Fig. 2c, right). While with buffer pre-stimulation, incubating the cells with the inhibitors had no significant effect on the PSC-RANTES-induced Ca^2+^ flux, with ATP pre-stimulation, the PSC-RANTES-induced Ca^2+^ flux is significantly decreased for cells incubated with all of the inhibitors as compared to the control cells incubated with just buffer. AR-C 118925XX and NF157 had a larger effect than the other inhibitors, decreasing the mean RFU by about twofold as compared with inhibitors such as apyrase and MRS2500, which only decreased the mean RFU by about 1.4-fold.

RANTES, the native CCR5 ligand, induces an increase in intracellular Ca^2+^ levels of 3830 RFU in the absence of inhibitors and with buffer pre-injection. This signal is not reduced upon incubation with inhibitors and the mean RFU values are similar for all conditions, except for the cells incubated with YM (Fig. 2d, left). ATP pre-stimulation vastly increases Ca^2+^ flux induced by RANTES by at least fourfold from 3830 RFU (buffer pre-stimulation) to 15,600 RFU (ATP pre-stimulation) (Fig. 2d, right). Similar to PSC-RANTES-induced Ca^2+^ flux, RANTES-induced Ca^2+^ flux was not inhibited by the purinergic receptor inhibitors with buffer pre-stimulation. However, with ATP pre-stimulation, the RANTES-induced Ca^2+^ flux is significantly decreased for cells incubated with all of the inhibitors as compared to the buffer control. All inhibitors reduced the increase in intracellular Ca^2+^ significantly by at least twofold (*p* < 0.0001).

ATPγS is an engineered version of ATP, in which one of the oxygens attached to 3-triphosphate is replaced by sulfur. This means that it is hydrolyzed very slowly by phosphatases and most ATPases. This serves as a control in our experiments, as the breakdown products of ATP, which can activate other purinergic receptors, will not be present. In the absence of any purinergic inhibitors (buffer control), ATPγS induces an increase in intracellular Ca^2+^ levels of 17,200 RFU for both buffer and ATP pre-stimulated cases (Fig. 2e). The pre-stimulation with ATP does not seem to affect the Ca^2+^ flux of cells stimulated by ATPγS, regardless of treatment with purinergic receptor inhibitors. The lack of reduced response suggests that perhaps breakdown products stimulating purinergic receptors are causing the second ATP injection to be reduced. In both ATP and buffer pre-stimulated cases, we see that incubation with apyrase, MRS2500, NF157, suramin, and YM cause a significant reduction in Ca^2+^ flux. The fact that apyrase has a significant reduction is noteworthy, since ATPγS should not be hydrolysable. However, apyrase is an ATP-diphosphohydrolase that catalyzes the sequential hydrolysis of ATP at the γ- and β-phosphates, so ATPγS could in fact, be a substrate for apyrase (Thomas et al. 1999). The reduction in Ca^2+^ flux upon incubation with apyrase can be explained by the breakdown of ATPγS, which means it can no longer stimulate the P2Y receptors. Overall, similar responses and inhibition are seen between ATP-induced Ca^2+^ flux and ATPγS-induced Ca^2+^ flux upon addition of purinergic receptor inhibitors, suggesting that the majority of the contribution to P2Y receptor-stimulated Ca^2+^ flux is from ATP and not its breakdown products.

Lastly, as a second control, we stimulated the CCR5-expressing cells with carbachol, which stimulates endogenous muscarinic receptors in HEK293T cells. Most muscarinic receptors are not significantly expressed in HEK293T cells, with the exception of muscarinic acetylcholine M3 receptors, which are the highest endogenously expressing muscarinic receptor in HEK293T cells (Atwood et al. 2011). They couple to Gq and so the increase in intracellular Ca^2+^ flux can be monitored in the same way (Fig. 2f). As expected, carbachol stimulates Ca^2+^ flux both in the absence and presence of purinergic receptor inhibitors, with all conditions showing a mean RFU of about 15,000. The ATP pre-stimulation increased the Ca^2+^ flux to about 22,000 RFU, but again, this was not significantly affected by incubation with the purinergic receptor inhibitors. The only significant decrease was seen when cells were incubated with YM, which would decouple the muscarinic M3 receptor from Gq. This confirms our hypothesis that the activation of purinergic receptors by ATP is only affecting CCR5-mediated Ca^2+^ flux and not the Ca^2+^ flux from other Gq-coupled receptors.

These results indicate that ATP pre-stimulation increases Ca^2+^ flux of CCR5-expressing cells stimulated by all second injection ligands, except for ATP and ATPγS. In particular, ATP pre-injection causes an enhancement in the increase in intracellular Ca^2+^ levels induced by PSC-RANTES and RANTES. We believe this is due to the activation of purinergic receptors by ATP, as this enhancement is reduced when the cells are incubated with purinergic receptor inhibitors. However, importantly, in the absence of an ATP pre-stimulation, the same inhibitors have no significant effect on PSC-RANTES- and RANTES-induced Ca^2+^ flux.

### The Difference in Ca^2+^ Flux of Cells With and Without ATP Pre-Stimulation Shows Largest Enhancement of RANTES-Induced Ca^2+^ Flux, Which Subsequently Shows Most Significant Reduction by Purinergic Receptor Inhibitors

**Fig. 3:**
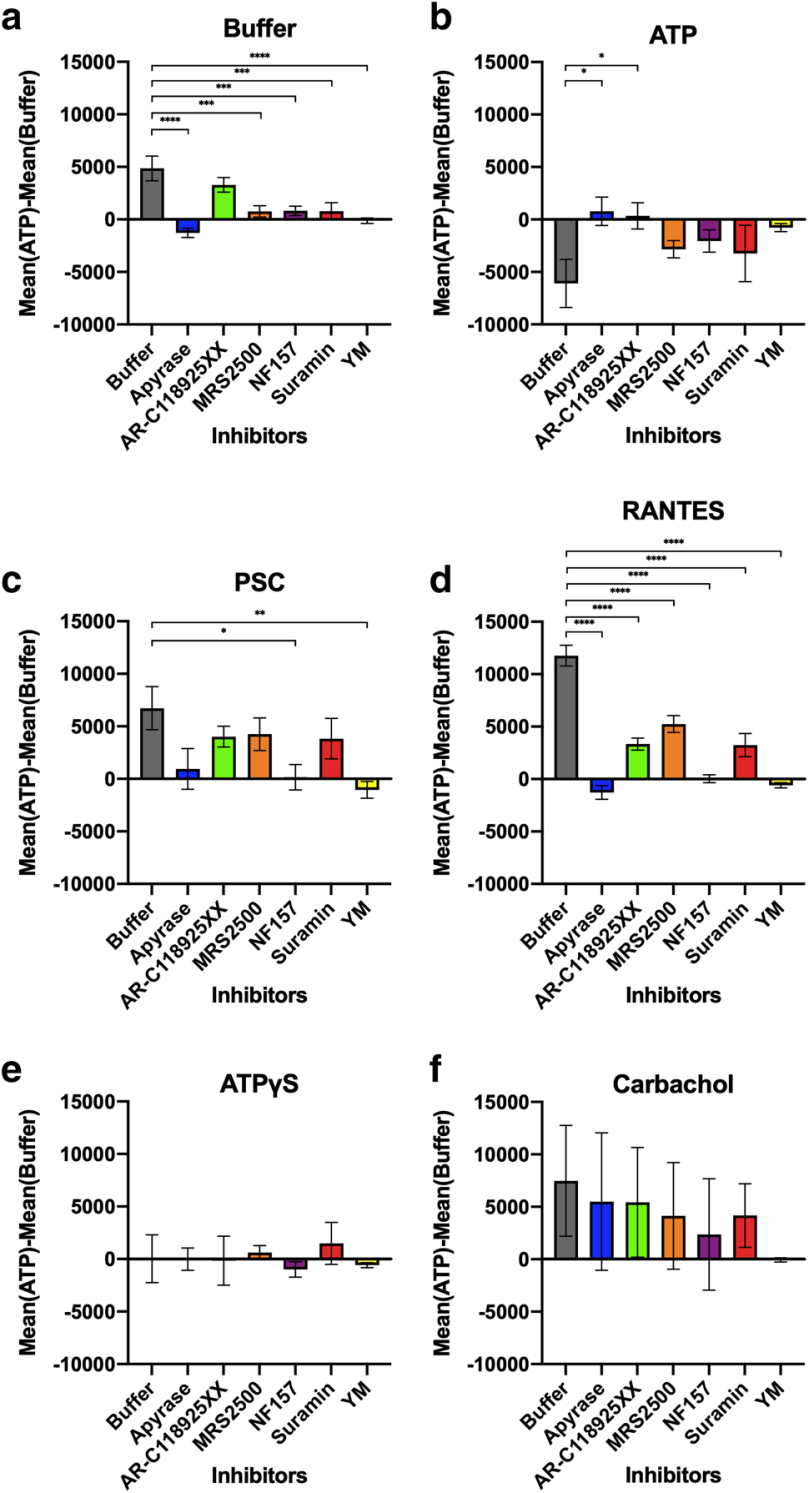
Difference in Ca^2+^ flux of cells with and without ATP pre-stimulation shows largest enhancement of RANTES-induced Ca^2+^ flux, which subsequently shows most significant reduction by purinergic receptor inhibitors. These graphs plot the difference in mean RFU of two sets of cells stimulated with six different ligands [**a** buffer, **b** ATP, **c** PSC, **d** RANTES, **e** ATPγS, and **f** carbachol], where one set of cells were pre-stimulated with 10 µM ATP (Fig. 2, right), and the other set of cells were not (buffer pre-injection, Fig. 2, left). All cells were incubated with various purinergic receptor inhibitors (listed in *x*-axis), as mentioned before. Each difference was compared to the difference obtained in buffer incubation case (gray). Dunnett’s multiple comparison test was used to assess significance of the ANOVA values and are shown above each bar. Data are mean ± SEM from three independent experiments with four technical replicates each. All ligands except ATP showed an increase in Ca^2+^ flux upon ATP pre-stimulation, whereas a double injection of ATP reduces the Ca^2+^ flux. ATP pre-stimulation showed large enhancements of PSC-RANTES and RANTES-induced Ca^2+^ flux, which subsequently showed the most significant reduction when incubated with purinergic receptor inhibitors

To better visualize the enhanced Ca^2+^ flux caused by ATP pre-stimulation, as well as the role of purinergic receptors, we calculated the difference in mean RFU of cells that were pre-stimulated with ATP and those that were not [mean RFU(ATP) − mean RFU(Buffer)]. This effectively shows the increase or decrease in Ca^2+^ flux caused by ATP pre-stimulation. For each ligand, the mean RFU(ATP) − mean RFU(Buffer) was compared with that of the control buffer incubation case (gray bar) and assessed for significance using a Dunnett’s multiple comparison test (Fig. 3).

As mentioned before, a buffer injection should not stimulate an increase in Ca^2+^ but due to the redistribution of ATP, the buffer injection following ATP pre-stimulation has a higher signal compared to the buffer injection following buffer pre-stimulation. This leads to a positive mean RFU(ATP) − mean RFU(Buffer) for the buffer control case (Fig. 3a). This positive difference is significantly decreased (*p* < 0.001 or 0.0001) when cells are incubated with all inhibitors except for AR-C 118925XX, the P2Y_2_ receptor blocker.

For ATP stimulation, the mean RFU(ATP) − mean RFU(Buffer) is negative for the buffer incubation control (Fig. 3b). This is due to desensitization of the receptors upon the first ATP injection, as mentioned earlier, which reduces Ca^2+^ flux from the second ATP injection. Apyrase, which hydrolyses ATP to ADP and AMP, serves as a good control, in which the mean RFU(ATP) − mean RFU(Buffer) becomes slightly positive and is significantly different from the buffer incubated cells (*p* < 0.1). The ATP from the pre-stimulation is digested by the apyrase and the receptors are not desensitized for the second injection. All other purinergic receptor inhibitors show a slightly less negative difference, as the effects of the ATP pre-stimulation are dampened, and receptor desensitization is not as prominent. It seems that the pre-stimulation of ATP does not affect the Ca^2+^ flux of cells stimulated by ATPγS, with most mean RFU(ATP) − mean RFU(Buffer) values hovering around zero (Fig. 3e). This suggests that perhaps breakdown products are causing the Ca^2+^ flux of the second ATP injection to be reduced and causing the negative mean RFU(ATP) − mean RFU(Buffer).

An ATP pre-stimulation moderately increases Ca^2+^ flux for CCR5-expressing cells stimulated with PSC-RANTES, and the mean RFU(ATP) − mean RFU(Buffer) is positive for the buffer incubation control (Fig. 3c). This positive value is significantly decreased when cells are incubated with NF157 and YM, such that the ATP pre-injection makes no difference to the Ca^2+^ flux stimulated by PSC-RANTES. NF157 inhibits P2Y_11_, which is the most highly expressed endogenous purinergic receptor in HEK293T cells (Table S1). Perhaps incubation with NF157 does not allow a substantial number of purinergic receptors to be stimulated by ATP in order to amplify CCR5-Ca^2+^ flux. If most of the Gq activation is through P2Y_11_ activation, incubation with the other inhibitors, which specifically inhibit other P2Y receptors, should show a smaller effect. This is reflected in our data. These data support the hypothesis that the enhancement in Ca^2+^ flux seen with ATP pre-stimulation is due to CCR5 cross-talking to purinergic receptors, particularly P2Y_11_.

The ATP pre-stimulation has the largest effect on CCR5-expressing cells stimulated with RANTES, leading to a great increase in Ca^2+^ flux and the largest difference in the mean RFU(ATP) − mean RFU(Buffer) for the buffer control (Fig. 3d). This large amplification is significantly decreased (*p* < 0.0001) upon incubation with all inhibitors, indicating that RANTES stimulation of CCR5-expressing cells is more sensitive to the purinergic receptor crosstalk. The activation of all purinergic receptors by ATP pre-stimulation is able to amplify the Ca^2+^ flux, as specifically inhibiting any one decreases the amplification. Similar to what was seen for PSC-RANTES, pre-incubation with NF157 essentially abolishes the effect of the ATP pre-stimulation, suggesting that P2Y_11_ is strongly involved in the crosstalk and Ca^2+^ flux amplification.

Lastly, ATP pre-stimulation has a slight increasing effect on CCR5-expressing cells stimulated with carbachol, leading to a positive mean RFU(ATP) − mean RFU(Buffer) for the buffer control (Fig. 3e). However, the error bars indicate that this increase is likely insignificant and further, there is no significant difference upon incubation with purinergic receptor inhibitors. Therefore, the pre-activation of purinergic receptors, which enhances PSC-RANTES- and RANTES-induced Ca^2+^ flux, is specifically affecting CCR5 and no other endogenous GPCRs.

These observations were also seen when the data corrected for second buffer injection, as outlined above, were used to calculate the mean RFU(ATP) − mean RFU(Buffer) (Fig. S3). Here, the purinergic receptor inhibitors all show a significant reduction of ATP-dependent enhancement of RANTES-stimulated Ca^2+^ flux, confirming that our most significant observations are not altered upon this correction. Here, as the data are being manipulated twice, the error accumulates and reduces the statistical significances when comparing to the buffer control data in a Dunnett’s multiple comparisons test. This may be why the significant reduction in the ATP-dependent enhancement of PSC-RANTES-stimulated Ca^2+^ flux by the purinergic receptor inhibitors was lost, although overall reduction trends look reproducible.

### RANTES-Stimulated CCR5-Ca^2+^ Flux is Completely Abolished by PTX and CGS15943

**Fig. 4:**
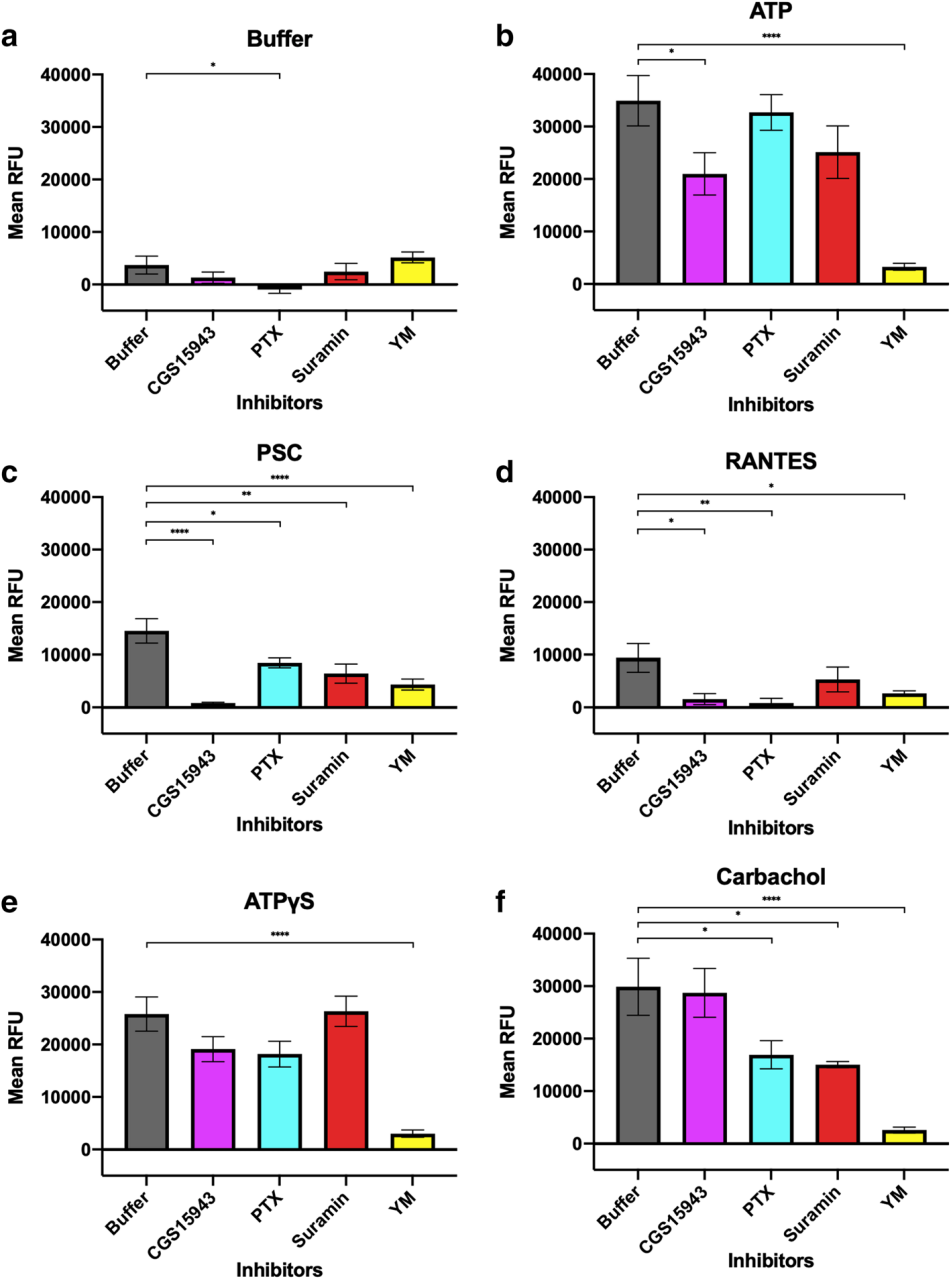
CCR5-expressing HEK293T cells were incubated with broadly acting inhibitors of G proteins and purinergic receptors. Similar experiments were conducted without the double injection, in which HEK293T cells expressing CCR5 were incubated with inhibitors in the *x*-axis and stimulated with one of six ligands: **a** buffer, **b** ATP, **c** PSC, **d** RANTES, **e** ATPγS, and **f** carbachol. The buffer incubation control shows the highest Ca^2+^ flux (gray). CGS15943, the broad adenosine receptor inhibitor (magenta), has a large reducing effect for both PSC-RANTES and RANTES. Pertussis toxin (PTX, cyan) abolishes RANTES-stimulated CCR5-mediated Ca^2+^ flux, but only moderately decreases PSC-RANTES-stimulated Ca^2+^ flux. YM (yellow) reduces both RANTES-stimulated and PSC-RANTES-stimulated Ca^2+^ flux. Suramin, the non-selective P2 purinergic inhibitor (red), reduced CCR5-mediated Ca^2+^ flux for both PSC-RANTES and RANTES to a similar degree. Data are mean ± SEM from two independent experiments with five and eight technical replicates each

We conducted similar experiments incubating CCR5-expressing cells with more broadly acting inhibitors of G proteins and purinergic receptors and stimulating with the same ligands, without the double injection (Fig. 4). Again, a buffer injection does not cause significant Ca^2+^ flux (Fig. 4a), while ATP injection causes the largest Ca^2+^ flux for the control case incubated in buffer, with a mean RFU of 35,000 (Fig. 4b). This is significantly reduced by CGS15943, the non-selective adenosine receptor inhibitor, as well as YM. ATPγS-stimulated Ca^2+^ flux is only affected by YM but not CGS15943 because there is no breakdown product, adenosine, to stimulate the adenosine receptors (Fig. 4e). CGS15943 also does not affect carbachol-stimulated Ca^2+^ flux (Fig. 4f), which is only reduced by the G protein blockers, YM and PTX, as well as suramin, the non-selective P2 purinergic receptor antagonists. This observation was surprising because muscarinic M3 receptor activation was not shown to be affected by purinergic receptors, but this reduction may be explained by the fact that suramin can also non-specifically block G protein coupling to GPCRs (Dunn and Blakeley 1988) (Table 1).

Interestingly, incubating cells with PTX, which inhibits Gi protein activation, completely abolishes RANTES-mediated CCR5-Ca^2+^ flux, but only decreases PSC-RANTES-mediated Ca^2+^ flux (Fig. 4c, d). On the other hand, the Gq inhibitor YM abolishes both RANTES- and PSC-RANTES-mediated CCR5-Ca^2+^ flux. This suggests that RANTES couples CCR5 to Gi, prior to the Gq-dependent Ca^2+^ flux through the P2Y receptors. However, PSC-RANTES couples CCR5 to Gq, which can undergo its own Ca^2+^ flux. Suramin, the non-selective P2 purinergic inhibitor, reduced CCR5-mediated Ca^2+^ flux stimulated by both PSC-RANTES and RANTES to similar degrees. CGS15943, the non-selective adenosine receptor inhibitor, seemed to have the largest reducing effect for both PSC-RANTES- and RANTES-induced Ca^2+^ flux. These data are unexpected, as this should still allow for purinergic receptors (P2Y and P2X receptors) to be activated by ATP, and so the reduction should not be as drastic. Are the adenosine receptors really playing a critical role in cross-talking with CCR5 to enhance PSC-RANTES- and RANTES-induced Ca^2+^ flux?

As adenosine is a late degradation product of ATP, we reasoned that it might accumulate in the media and cause the large effect of the broad-spectrum adenosine receptor, CGS15943. To rule out any effects from the accumulation of ATP and its breakdown products within the assay wells, we would want to wash the cells prior to Ca^2+^ flux measurement. The FLIPR Calcium 6 dye uses “masking technology”, in which the Ca^2+^ dye is added to the cells alongside a loading buffer, which contains chemicals that reduce background fluorescence originating from residual extracellular Ca^2+^ indicator, media, and other components. Loading buffers may be hypertonic, which creates undefinable and uncontrolled conditions within the wells from dead or lysed cells due to this change is osmotic potential (Zlokarnik 2000). As the loading buffer is necessary for taking the Ca^2+^ flux measurements, we cannot easily wash the cells either. To address these concerns, we optimized experiments using a different method to take Ca^2+^ flux measurements, using a genetically encoded Ca^2+^ indicator, GCaMP6s. GCaMP6s consists of a circularly permuted green fluorescent protein (cpGFP), the Ca^2+^-binding protein calmodulin (CaM), and CaM-interacting M13 peptide with a chromophore within the cpGFP β barrel. Upon CaM binding to Ca^2+^, there are Ca^2+^-dependent conformational changes causing increased brightness of the chromophore which can be conveniently monitored at the same wavelengths as those of the FLIPR Calcium 6 dye (Chen et al. 2013). This system does not involve the loading buffer associated with the FLIPR Calcium 6 dye and allows for media change right before Ca^2+^ flux measurement. Genetically encoded Ca^2+^ sensors often suffer from lower sensitivity and signals as compared to Ca^2+^ dyes, so we repeated the double injection experiments introduced in Figs. 2, 3, and S1 to see if we could reproduce these data. The resulting data are shown in Figures S4, S5, and S6. The GCaMP6s experiments led to signals that were about fivefold lower than in the FLIPR Calcium 6 experiments. For example, ATP-stimulated Ca^2+^ flux incubated in the buffer control gave a mean RFU of 17,000 with FLIPR Calcium 6 dye but only 3200 with GCaMP6s. Nonetheless, the double injection experiments with GCaMP6s confirm the overall findings from the FLIPR data sets, leading to the same trends caused by ATP pre-stimulation and purinergic receptor inhibitor incubation. However, since the signals are much weaker in the GCaMP6s system, some statistically significant differences are lost or reduced.

### Washing the CCR5-Expressing Cells Prior to Ca^2+^ Flux Measurement Abolishes the Large Reduction in RANTES-Stimulated Ca^2+^ Flux Caused by CGS15943 but does not Affect the Reduction Caused by the P2Y Receptor Inhibitors

As we have shown that the GCaMP6s system works well in our hands to reproduce the effects seen from the purinergic receptor inhibitors on CCR5-mediated Ca^2+^ flux, we went on to investigate the large effect of CGS15943 further. In order to do this, we took advantage of the fact that the GCaMP6s system allows us to wash the cells prior to Ca^2+^ flux measurement, as well as the fact that no hypertonic loading buffers need to be used. Here, HEK293T cells were co-transfected with CCR5 and GCaMP6s. As before, the cells were incubated with broadly acting inhibitors of G proteins and purinergic receptors and stimulated with ligand. Half of the cells were washed prior to reading the Ca^2+^ flux, removing any old media that may contain dead cells, ATP, and fetal bovine serum (FBS) which contributes adenosine to the wells.

We were concerned that aspiration and replacement of the media in a 384-well may lead to cell loss, which may affect the Ca^2+^ flux signals upon ligand injection. To account for this, we quantified the cell loss by once again, using the double injection feature of the FlexStation. We injected 10 µL of 0.1% Triton X-100 (final conc.) after the cells were stimulated by their respective ligands. This lyses the cells and releases GCaMP6s, which causes a measurable increase in GFP fluorescence as they come into contact with free extracellular Ca^2+^. In this way, the corrected mean RFU signals from the second injections quantify the total cell count in the washed or non-washed wells (Fig. S7). The wash step reduced the fluorescence from 6400 RFU (non-washed) to 5800 RFU (washed) indicating about a 9% cell loss.

**Fig. 5:**
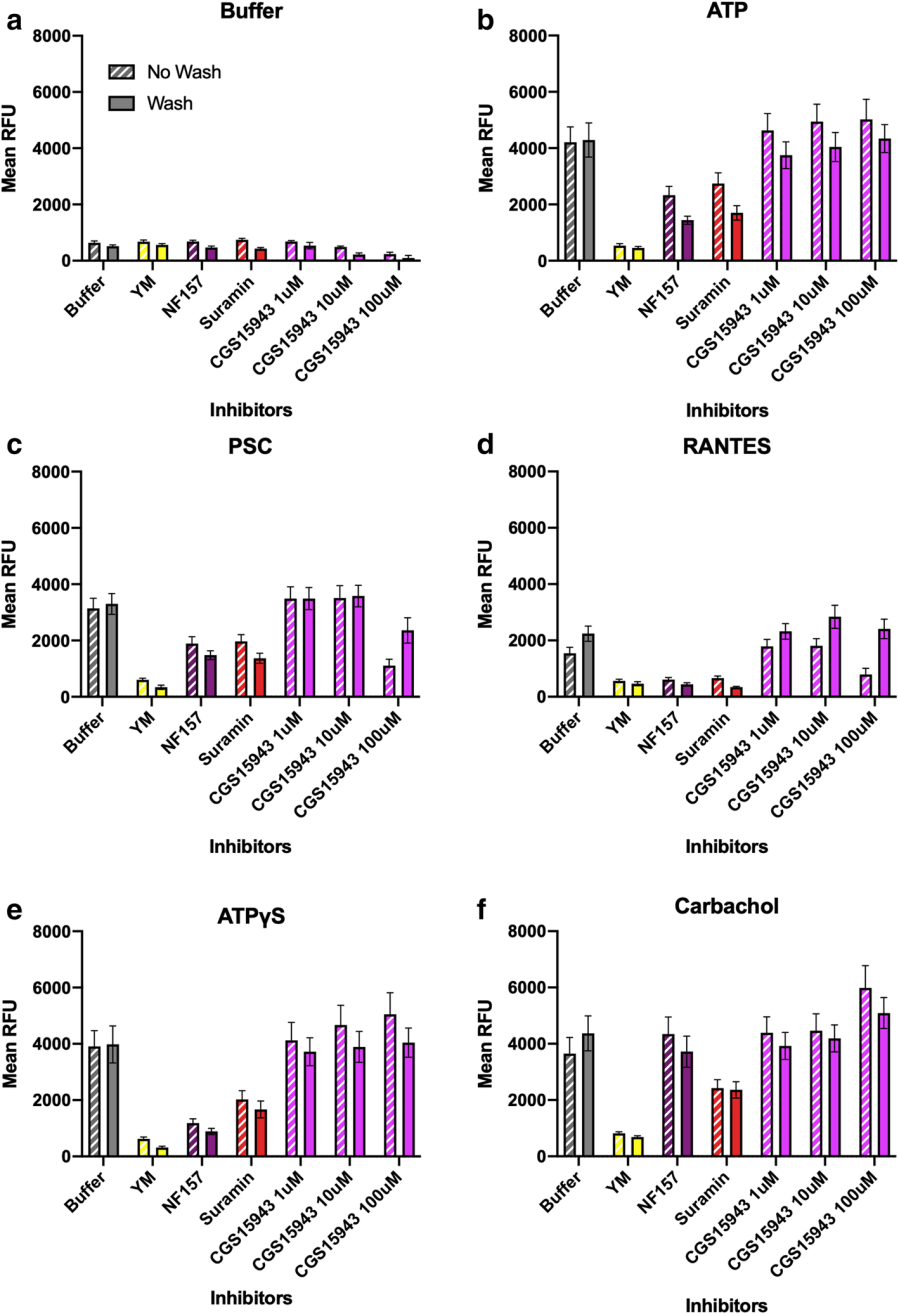
Washing the CCR5-expressing cells prior to Ca^2+^ flux measurement abolishes the large reduction in RANTES-stimulated Ca^2+^ flux caused by CGS15943 but does not affect the reduction caused by the P2Y receptor inhibitors. Similar experiments as those presented in Fig. 4 were conducted using GCaMP6s, a genetically encoded protein Ca^2+^ sensor, in place of the FLIPR Calcium 6 dye. Here, HEK293T cells were co-transfected with CCR5 and GCaMP6s. As before, the cells were incubated with inhibitors listed in the *x*-axis and stimulated with one of six ligands: **a** buffer, **b** ATP, **c** PSC, **d** RANTES, **e** ATPγS, and **f** carbachol. Half of the cells were washed prior to reading the Ca^2+^ flux, removing any old media which may contain dead cells as well as ATP, its breakdown products, and adenosine from FBS (solid bars). Data from wells that were not washed are shown in the striped bars. CGS15943 caused a large reduction in PSC-RANTES- and RANTES-stimulated Ca^2+^ flux in the FLIPR Calcium 6 assay (Fig. 4) but we see here that this is largely abolished upon cell washing (solid, magenta bars). On the other hand, the P2Y receptor blockers (suramin, red; NF157, purple) are not affected by media exchange. Data are mean ± SEM from three independent experiments with three or four technical replicates each

For cells stimulated with ATP, ATPγS, and carbachol, washing the cells reduced the Ca^2+^ flux of cells incubated with the inhibitors, as compared to those that were not washed (Fig. 5b, e, f). The average reduction in Ca^2+^ flux for the washed wells as compared to the non-washed wells was about 10%, which can be explained by the ~ 9% cell loss quantified earlier. However, when we focus on PSC-RANTES- and RANTES-stimulated Ca^2+^ flux, we see that the large inhibitory effect of CGS15943 from Fig. 4 is largely abolished upon cell washing (solid magenta bars, Fig. 5c, d). Without washing (striped magenta bars), a significant decrease in PSC-RANTES- and RANTES-stimulated Ca^2+^ flux is seen at the highest concentration of CGS15943, 100 µM, which is what was used in the previous experiment. However, when cells are washed, even at the highest concentration, there is no reduction seen in RANTES-stimulated Ca^2+^ flux and only a slight reduction in PSC-RANTES-stimulated Ca^2+^ flux. This effect is very different from the dramatic reductions we observed with the FLIPR Calcium 6 dye, as well as the decreases seen in the unwashed cells at 100 µM of CGS15943. On the other hand, the effects of the P2Y receptor blockers (suramin, red; NF157, purple) are not affected by media exchange. This confirms our hypothesis that PSC-RANTES- and RANTES-stimulated CCR5 are cross-talking predominantly with P2Y receptors and not with the adenosine receptors. The large reduction from before may have been a combined effect of the FLIPR Calcium 6 loading buffer and accumulation of ATP, FBS and adenosine in the assay wells. We suspect that there may also be a non-specific effect on CCR5 at the highest concentrations of CGS15943, as the PSC-RANTES- and RANTES-stimulated Ca^2+^ fluxes are both reduced drastically when wells are not washed (100 µM is ~ 10,000 × IC_50_ of CGS15943, Table 1).

## Discussion

In a similar way in which ATP activation of endogenous P2Y receptor in CCR4-CHO cells enabled an otherwise absent Ca^2+^ response to be elicited by the chemokines MDC and TARC, there seems to be crosstalk between the native P2Y receptors and the transfected CCR5 receptors in the HEK293T cells (Rosethorne et al. 2004). We show here that in HEK293T cells expressing CCR5, RANTES, and PSC-RANTES to a certain extent, showed a large Ca^2+^ flux amplification upon ATP pre-stimulation. Incubating cells with PTX, which inhibits Gi protein activation, completely abolished RANTES-mediated CCR5-Ca^2+^ flux, but only decreased PSC-RANTES-mediated Ca^2+^ flux. The Gq inhibitor YM, on the other hand, abolished both RANTES- and PSC-RANTES-mediated CCR5-Ca^2+^ flux. These data mirror what was found previously, where YM knocked out both RANTES- and PSC-RANTES-mediated CCR5-Ca^2+^ flux. However, because PSC-RANTES biases CCR5 coupling toward Gq while RANTES biases CCR5 coupling toward Gi, PTX only abolishes stimulation from RANTES and not PSC-RANTES (Lorenzen et al. 2018).

It follows that RANTES-stimulated CCR5-Ca^2+^ flux showed the largest amplification by ATP pre-stimulation, because RANTES couples the receptor to Gi. This means that the Ca^2+^ flux is a result of Gβγ subunit release. It is thought that PLC-β binds to the βγ subunit at the N-terminal tail and the Gαq subunit at the C-terminal tail, downstream of the Y domain, at distinct sites. This means that both can bind at the same time, and it is predicted that occupancy of one site may modulate efficacy of the second. In particular, Gαq bound to PLC increases efficacy of βγ (Smrcka and Sternweis 1993). Accordingly, when the P2Y purinergic receptors are stimulated, they activate Gq, which can modulate PLC-β affinity for the βγ subunit from CCR5-activated Gi. This is supported by our observation that the ATP pre-stimulation did not increase RANTES-stimulated Ca^2+^ flux when cells were incubated with purinergic receptor inhibitors. This is because P2Y purinergic receptors were no longer activated and the crosstalk, by which Gαq binding to PLC-β increases the βγ affinity of PLC-β, was abolished. This crosstalk is so sensitive that inhibiting any specific P2Y receptor decreased the amplification. Importantly, through our careful analysis using the GCaMP6s system and washing the cells prior to Ca^2+^ flux measurement, we confirmed that RANTES-stimulated CCR5 is cross-talking predominantly with P2Y receptors and not the adenosine receptors. ATP is the crucial ligand stimulating the purinergic receptors to enhance RANTES-stimulated Ca^2+^ flux and the breakdown product, adenosine, stimulating adenosine receptors does not play a role in this enhancement. In a slight aside, this experiment underlines the importance of considering the side-effects of the FLIPR Calcium 6 loading dye and the inability to control the degradation products and other contents in the assay wells. The ability to wash the cells prior to Ca^2+^ flux measurement highlights the advantages of the GCaMP6s system, albeit the lower signals.

PSC-RANTES may not be as sensitive to this enhancement because it couples CCR5 to Gq effectively. There is still an amplification from the activated P2Y receptors, but the pre-injection of ATP does not have as pronounced of an effect. Cells incubated with AR-C 118925XX, MRS2500, and suramin do not show a significant decrease in the Ca^2+^ amplification, although they did for RANTES-stimulated cells. When only some of the P2Y receptors are inhibited, there is not a marked effect. The exception to this was when cells were incubated with NF157, which blocks P2Y_11_. P2Y_11_ is the most highly expressed endogenous purinergic receptor in HEK293T cells (Table S1), and unsurprisingly, caused the most significant loss in ATP pre-injection mediated Ca^2+^ flux amplification.

A recent study showed that the Gs-coupled β_2_-adrenergic receptors (β_2_AR) transactivated Gq-coupled purinergic receptors and increased intracellular Ca^2+^ upon activation of β_2_AR by agonist isoproterenol (ISO) (Stallaert et al. 2017). The authors incubated a HA-β_2_AR-HEK293S stable cell line with purinergic receptor inhibitors, analogous to our work. They chose the inhibitors for the most highly expressed receptors, P2X_1_, P2X_4_, P2X_7_ and P2Y_11_, as well as CGS15943 and suramin. Interestingly, CGS15943 and the inhibitors for the P2X receptors showed no effect on the ISO-promoted increase in intracellular Ca^2+^. NF157 and NF340, which both block P2Y_11_, significantly reduced the increase in Ca^2+^. Furthermore, incubation with apyrase decreased both the efficacy (decrease in *E*_max_) and potency (increase in EC_50_) of ISO-promoted Ca^2+^ increase. This means that the extracellular mediator, ATP, released upon β_2_AR activation, is depleted and no longer able to transactivate P2Y_11_. Although we did not test any inhibitors of the P2X receptors, we similarly found that the highly expressed P2Y_11_ was predominantly responsible for the increase in intracellular Ca^2+^ and incubation with both NF157 and apyrase abolished the increase in intracellular Ca^2+^. This example of purinergic receptor crosstalk with a GPCR is slightly different from our example, however, as β_2_AR-mediated release of ATP stimulates P2Y_11_ to invoke a Ca^2+^ flux when there otherwise is none. In our example, chemokine-stimulated CCR5 induces Ca^2+^ flux, but there is an enhancement when ATP first stimulates P2Y_11_. Together with our results, these findings demonstrate that both Gs- and Gi-coupled GPCRs can crosstalk with Gq-coupled purinergic receptors activated by ATP. We speculate that this crosstalk could be the underlying mechanism of the reported off-target effects of YM and FR900359 on Gs and Gi signaling (Gao and Jacobson 2016; Peng and Shen 2019), rather than a direct interaction of these Gq-inhibitors with Gs or Gi proteins.

**Scheme 1:**
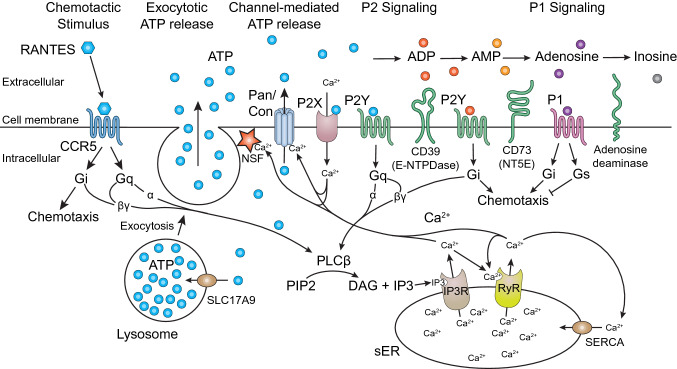
Purinergic amplifier model for CCR5. CCR5 works in concert with P2X and P2Y purinergic receptors to amplify each other’s responses. CCR5 is stimulated extracellularly by the chemokine RANTES and is coupled to both Gq and Gi proteins. PLC-β is the classical effector of Gq but can also be activated by the βγ subunits from both G protein families. PLC-β hydrolyses PIP2 into IP3, which bind to and activate IP3 receptors to release Ca^2+^ stored in the sER. Exocytotic ATP release via NSF and channel-mediated ATP release are both Ca^2+^ dependent, and thus CCR5 and PLC-β activation provide the substrates for the purinergic receptors to the extracellular side. Completing this circle, P2Y purinergic receptors at the cell surface are activated by ATP and couple to Gq and Gi. As indicated before, PLC-β can be activated by the α subunit of the Gq protein or the βγ subunits from both Gq and Gi proteins so the purinergic receptors can crosstalk with CCR5 and enhance its response

We propose a model in which the CCR5 and P2X and P2Y purinergic receptors work in a mutually beneficial way to amplify each other’s responses (Scheme 1). CCR5 is coupled to both Gq and Gi proteins, which, upon activation dissociate from the receptor and into their constituent subunits to interact with downstream signaling enzymes. PLC-β is the classical effector of the α subunit of Gq but can also be activated by the βγ subunits from both G protein families. PLC-β hydrolyses phosphatidylinositol 4,5-bisphosphate (PIP2) into inositol 1,4,5-trisphosphate (IP3), which bind to and activate IP3 receptors, which act as Ca^2+^ channels to release Ca^2+^ stored in the smooth endoplasmic reticulum (sER) (de Rubio et al. 2018). Other than the IP3 receptors, the ryanodine receptors (RyR) are also involved in the release of Ca^2+^ from the sER, but HEK293T cells do not endogenously express RyR, so the flux we are seeing can be attributed wholly to the IP3 receptors (Tong et al. 1999). Sarco/endoplasmic reticulum Ca^2+^-ATPase (SERCA) pump Ca^2+^ into the sER from the cytosol to maintain the high concentration of Ca^2+^ inside. Once the cytosolic concentrations of Ca^2+^ increase, there is a cascade of intracellular activity. However, an important point is that Ca^2+^ is also required for the release of ATP to the extracellular side. *N*-ethylmaleimide-sensitive factor (NSF) is a AAA ATPase involved in fusion of the ATP-rich lysosome to the cell membrane, and the exocytotic ATP release is Ca^2+^ dependent (Cao et al. 2014; Sivaramakrishnan et al. 2012; Zhao and Brunger 2016). Moreover, there are Ca^2+^ dependent ATP-channels, which release ATP to the extracellular side as well.

Thus, CCR5 and PLC-β activation mobilizes ATP and its degradation products, the substrates for the purinergic receptors, to the extracellular side relevant for receptor activation. The P2X receptor, which is a cation-permeable ligand-gated ion channel, opens in response to the binding of extracellular ATP and lets Ca^2+^ back inside to mediate further ATP release. Every cell is capable of Ca^2+^ dependent release of lysosomal contents, which is essentially a one molar solution of ATP (Zhang et al. 2007). This autocrine purinergic signal is able to amplify the cellular Ca^2+^ signal by the ubiquitous presence of P2Y purinergic receptors at the cell surface, many Gq coupled (Corriden and Insel 2010). As indicated before, PLC-β can be activated by the α subunit of the Gq protein or the βγ subunits from both Gq and Gi proteins, so the α subunit from P2Y binds to PLC-β and primes it for subsequent activation by the Gβγ subunit from chemokine receptor-activated Gi, or vice versa. Ultimately, the crosstalk between CCR5 and the purinergic receptors enhances the CCR5 response. The CCR5 pathway in turn supplies ATP for purinergic receptor activation, and thus the two receptors acting in concert creates an amplifier circuit.

There is also a second mechanism of purinergic amplification. The second mechanism is from secretory release of GPCR ligands (chemokines, opioids, monoamines, acetylcholine, etc*.*) from secretory vesicles, which are full of ATP in the same way lysosomes are, and so their release leads to a concurrent liberation of ATP (Estévez-Herrera et al. 2016). As described previously, upon chemokine-stimulated Ca^2+^ flux in the cells, lysosomes fuse to the membrane in a localized area. This leads to a local increase in the ATP concentration upon release of the lysosomal contents. Having two simultaneous local diffusible signals leads to a strong local activation of Ca^2+^ signaling in target cells. These two signals rapidly dissipate by either diffusion, enzymatic degradation (by metalloproteases or cholinesterases) or reuptake (monoamines). Thus, synergistic signaling of the two components will dramatically sharpen the precision of the local signal, which is essential for chemotaxis and also for neurotransmission.

It is very challenging to distinguish between these two modes of ATP increase experimentally. In our current experiments, we are inhibiting P2Y receptors, but this still allows both lysosomal release of ATP, as well simultaneous release of chemokines and ATP from secretory vesicles, to occur. In a similar way that purinergic receptor inhibitors reduce the RANTES-stimulated Ca^2+^ flux, inhibitors of the lysosomic pathway should also reduce RANTES-stimulated Ca^2+^ flux by blocking one of the two modes of ATP increase. However, since they will not be inhibiting both modes of ATP increase, we cannot completely inhibit the crosstalk and thus we will not be able to adequately validate our model. We will keep exploring experimental techniques in future follow up studies to convincingly validate the purinergic amplifier model.

More broadly, the binding of Gαq to a PLC-β isoform (possibly PLC-β3) is thought to increase its affinity for the βγ subunits derived from activated δ-opioid receptors in NG108-15 cells, due to a relief of steric hindrance (Yoon et al. 1999). Another study showed that the βγ subunits from the δ-opioid receptor-activated Gi proteins played a key role in Ca^2+^ flux by overexpressing the βγ-binding domain of G protein-coupled receptor kinase 2 (GRK2) and sequestering free βγ subunits (Yeo et al. 2001). This abolished Ca^2+^ flux, but while crucial, they are not sufficient to activate PLC-β alone, so the synergistic activation of PLC-β by activation of Gq-coupled M3 muscarinic receptors is a plausible hypothesis, parallel to what we have seen here with CCR5 and P2Y receptors. It has been known that ATP is released along with neurotransmitters from synaptic vesicles, but its role is varied depending on each system (Holton 1959). ATP has been shown to be a co-transmitter with noradrenaline (NA), 5-hydroxytryptamine, glutamate, dopamine, and g-amino butyric acid (GABA) in the central nervous system (CNS) (Burnstock 2006). Peptidergic neurotransmitters, such as endogenous opioids, are stored in dense core vesicles (DCVs) in neurons, which are larger than the vesicles which carry small-molecule neurotransmitters, but have been shown to store ATP as well (Jekely et al. 2018). The synthesis, sorting, and trafficking of neuropeptides involves many distinct enzymes and steps, making them expensive agonists for the cell to produce. As δ-opioid receptors require ATP pre-stimulation of the P2 purinergic receptors to signal, one can speculate that ATP can act as an inexpensive extracellular mediator that is co-transmitted with the neuropeptides to boost the Ca^2+^ flux response. Although the mechanism is not yet clear, it is evident that pre-stimulating with ATP and activating the purinergic receptors enhances the Ca^2+^ response of Gi-coupled receptors like CCR5. To this end, ATP could play an important role in amplifying the responses of costly peptide-activation (such as by chemokines and endogenous opioids) by acting as an inexpensive intermediary agonist to boost intracellular Ca^2+^ signaling via crosstalk with purinergic receptors.

In conclusion, we have found that Ca^2+^ flux of CCR5-expressing HEK293T cells stimulated by RANTES, and to some extent PSC-RANTES, can be primed and enhanced by a prior stimulation of ATP. This enhancement comes from the crosstalk between the Gi-coupled CCR5 and endogenous Gq-coupled P2Y receptors, particularly the P2Y_11_ receptor, which is able to enhance the PLC-β activity downstream. This amplification occurs via two mechanisms of ATP release. First, there is the agonist-dependent secretory release of lysosomal contents and second, there is the secretory release of GPCR ligands (chemokines, opioids, monoamines, acetylcholine, etc*.*) from secretory vesicles. Both methods of ATP release allow for crosstalk between ATP-activated purinergic receptors and Gi-coupled GPCRs to amplify the intracellular Ca^2+^ signaling response.

## Electronic supplementary material

Below is the link to the electronic supplementary material.

Electronic supplementary material 1 (DOCX 3166 kb)

## Data Availability

All data needed to evaluate the conclusions in the paper are presented in the paper or the Supporting Information (SI). Additional requests should be made to the corresponding author.
