# Supplementary material for: Purinergic Receptors Crosstalk with CCR5 to Amplify Ca2+ Signaling
Source: Cell Mol Neurobiol. 2020 Nov 20;41(5):1085–101. doi: 10.1007/s10571-020-01002-1 (PMC8159800; doi:10.1007/s10571-020-01002-1)
Supplement: Supplementary file 1 — Electronic supplementary material 1 (DOCX 3166 kb) [file 10571_2020_1002_MOESM1_ESM.docx]

Cellular and Molecular Neurobiology

**Purinergic Receptors Crosstalk with CCR5 to Amplify Ca^2+^ Signaling**

Mizuho Horioka,^1,2^ Emilie Ceraudo,^2^ Emily Lorenzen,^2^ Thomas P. Sakmar^2,^* and Thomas Huber^2,^*

^1^Tri-Institutional Program in Chemical Biology, New York, NY 10065, USA.

^2^Laboratory of Chemical Biology and Signal Transduction, The Rockefeller University, 1230 York Ave., New York, NY, USA

*Correspondence: T.P.S. sakmar@rockefeller.edu, T.H. hubert@rockefeller.edu

**Supplemental Information**

**Fig. S1** **ATP stimulation causes an increase in Ca^2+^ flux in CCR5-expressing HEK293T cells that is reduced when incubated with purinergic receptor inhibitors**

The increase in intracellular Ca^2+^ levels were monitored upon stimulation of HEK293T cells transiently transfected with CCR5 and incubated with purinergic receptor inhibitors (listed in x-axis). These data show the first injection, which was either buffer or 10 µM ATP. The corrected mean RFU for this first injection was calculated from the raw data (t_2_− t_1_) as shown in **Fig. 1**. As expected, the buffer injection (black bars) did not give a significant increase in mean RFU. The ATP injection induces Ca^2+^ flux (gray bars), which is decreased by differing amounts upon incubation with the purinergic receptor inhibitors. Data are mean ± SEM from three independent experiments with 22 technical replicates each, averaged over all wells that have different second injection ligands.

**
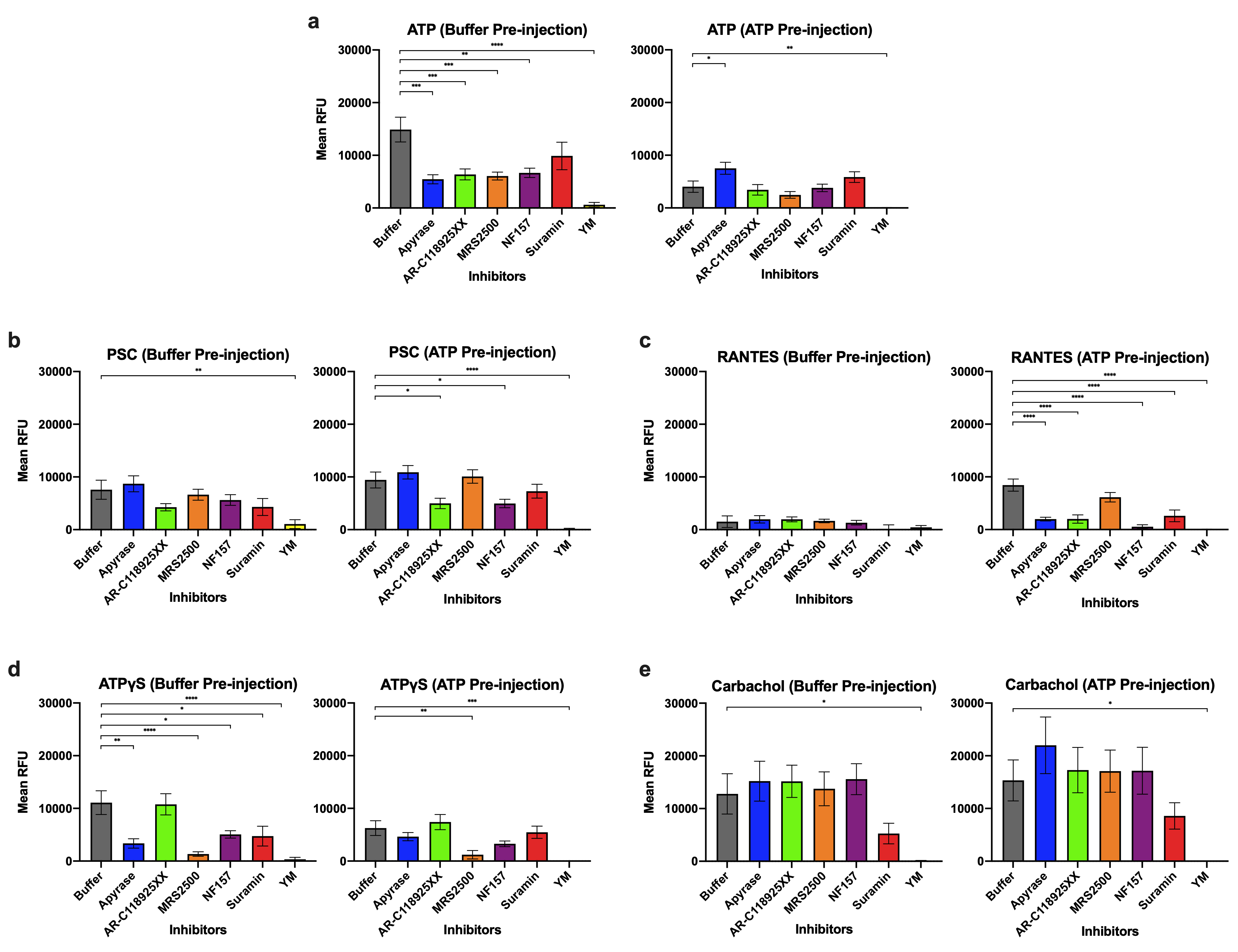
**

**Fig. S2 Correcting for the ATP redistribution caused by the second injection reproduces the trends seen pre-correction in Fig. 2**

The redistribution of ATP from the pre-injection causes the second injection of buffer to stimulate a significant increase in Ca^2+^ flux (**Fig. 2a, right**). As this redistribution effect will be present for all second injections, we corrected for this effect by treating the second buffer injection as background signal. Thus, the corrected mean RFU for the second buffer injection (t_4_− t_3_) was subtracted from the respective mean RFU signals from second injections of all other ligands incubated with the same inhibitor. This was done for both buffer and ATP pre-injection signals and the results are plotted here. The major trends for each ligand are reproducible compared to those pre-correction in **Fig. 2**.

**
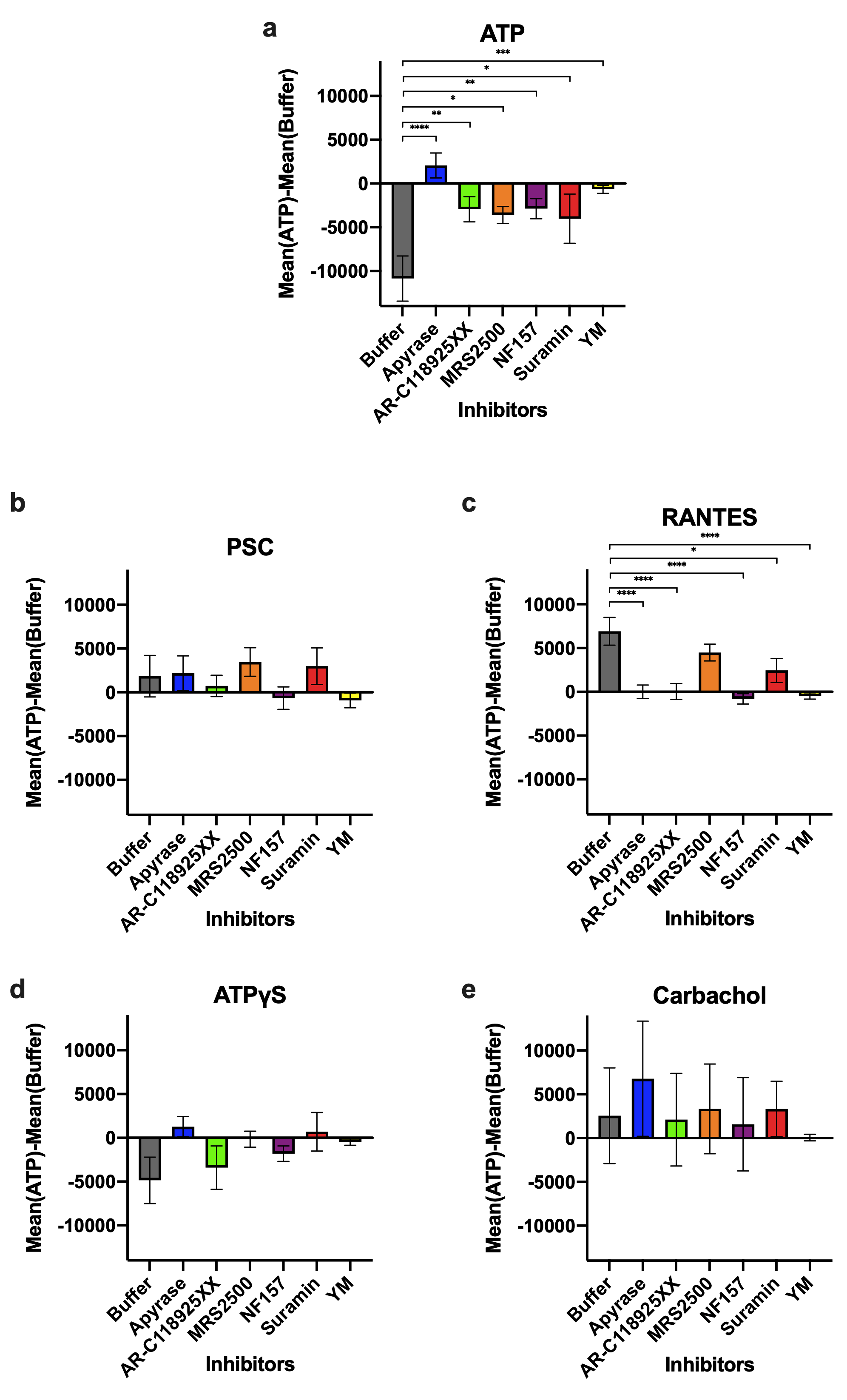
**

**Fig. S3 Data corrected for second buffer injection confirms the significant reduction of ATP-dependent enhancement of RANTES-stimulated Ca^2+^ flux by purinergic receptor inhibitors**

The corrected RFU values for the second injections were used to plot the difference in mean RFU of cells pre-injected with 10 µM ATP and cells pre-injected with buffer. Again, the main observations from **Fig. 3** are confirmed and the ATP-dependent enhancement of RANTES-stimulated Ca^2+^ flux was largely and significantly reduced by incubation with purinergic receptor inhibitors. As the data are being manipulated twice, the error accumulates and reduces the statistical significances of the data. This may be why incubation with purinergic receptor inhibitors did not significantly reduce the ATP-dependent enhancement of PSC-RANTES-stimulated Ca^2+^ flux and the data should be reserved for the supplement.
**Fig. S4 GCaMP6s captures reduction of ATP stimulated-CCR5 Ca^2+^ flux caused by purinergic receptor inhibitors as seen in FLIPR Calcium 6 assay**

Similar experiments as those in **Fig. S1** were conducted using GCaMP6s, a genetically encoded protein Ca^2+^ sensor, in place of the FLIPR Calcium 6 assay. Here, HEK293T cells were co-transfected with CCR5 and GCaMP6s. As before, the cells were incubated with inhibitors listed in the x-axis and stimulated with 10 µM ATP (gray) or buffer (black). Although the mean RFU signals are lower, the GCaMP6s Ca^2+^ sensor shows the same effect of the purinergic receptor inhibitors on ATP-stimulated CCR5 Ca^2+^ flux as seen in **Fig. S1** using the FLIPR Calcium 6 assay. Data are mean ± SEM from two independent experiments with 22 technical replicates each, averaged over all wells that have different second injection ligands.

**
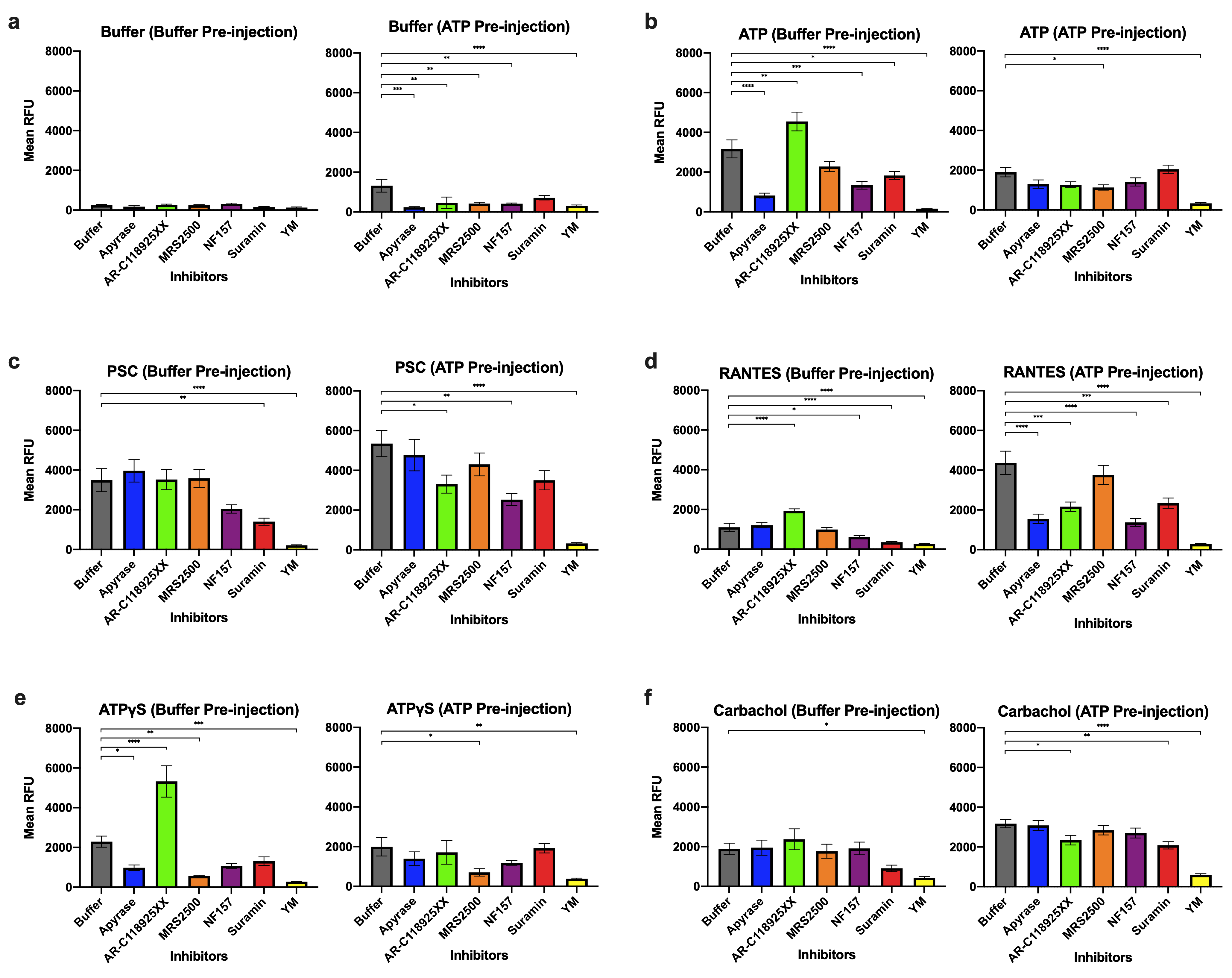
Fig. S5 Parallel double injection experiments with GCaMP6s Ca^2+^ sensor confirm the findings from the FLIPR Calcium 6 experiments in Fig. 2**

CCR5 and GCaMP6s-encoding HEK293T cells that were pre-injected with buffer (left) or 10 µM ATP (right), followed by a second injection of one of six ligands: **a** buffer, **b** ATP, **c** PSC, **d** RANTES, **e** ATPγS, and **f** carbachol. Prior to injection, cells were incubated with purinergic receptor inhibitors for 30 mins (2 hrs for YM), which are listed in the x-axis. Each mean RFU was compared to the mean RFU of the buffer incubation case (control, gray). Dunnett’s multiple comparison test was used to assess significance of the ANOVA values and are shown above each bar. Data are mean ± SEM from two independent experiments with four technical replicates each. All inhibitors show a similar effect on ligand-induced Ca^2+^ flux as seen in **Fig. 2**, confirming that the findings from the FLIPR Calcium 6 data can be replicated using a different mode of detecting and quantifying Ca^2+^ flux. The signals are lower overall as compared with those obtained using FLIPR Calcium 6 in **Fig. 2.**

**
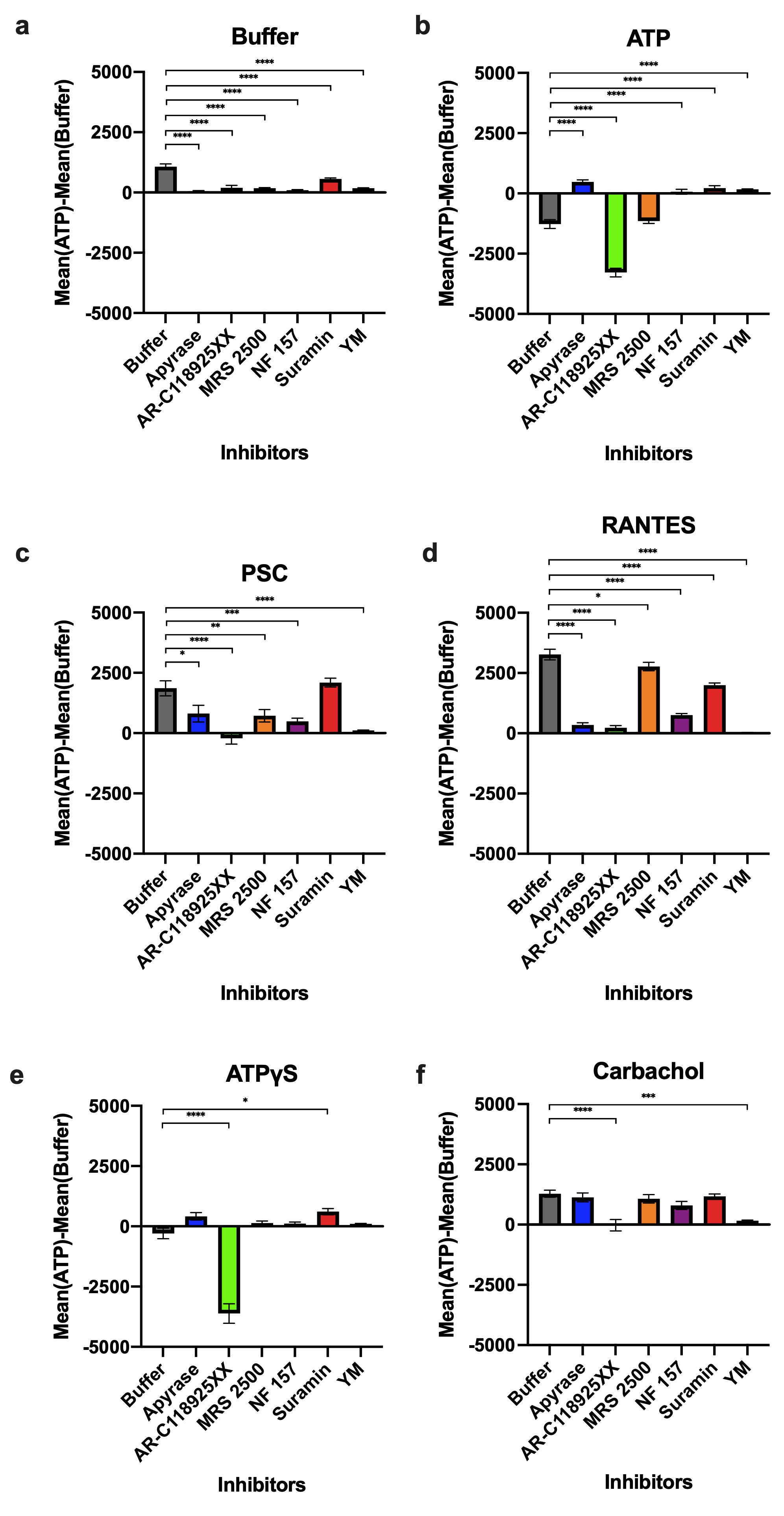
**

**Fig. S6 Parallel experiments conducted with GCaMP6s Ca^2+^ sensor confirm the reduction of ATP-dependent enhancement of PSC-RANTES- and RANTES-stimulated Ca^2+^ flux by purinergic receptor inhibitors**

These graphs plot the difference in mean RFU of two sets of cells co-transfected with GCaMP6s and CCR5, stimulated with six different ligands [**a** buffer, **b** ATP, **c** PSC, **d** RANTES, **e** ATPγS, and **f** carbachol], where one set of cells were pre-stimulated with 10 µM ATP (**Fig. S5, right**), and the other set of cells were not (buffer pre-injection, **Fig. S5, left**). All cells were incubated with various purinergic receptor inhibitors (listed in x-axis), as mentioned previously. Each difference was compared to the difference obtained in the control, buffer incubation case (gray). Dunnett’s multiple comparison test was used to assess significance of the ANOVA values and are shown above each bar. Data are mean ± SEM from two independent experiments with four technical replicates each. Although the statistical significances of the differences are lost or reduced due to the weaker signals of the GCaMP6s system, the general trends in reduction caused by each purinergic receptor inhibitor remain the same.

**Fig. S7 Washing and replacing of media prior to Ca^2+^ flux measurement causes cell loss**

Following the experiment in **Fig. 5**, where half of the wells containing CCR5 and GCaMP6s-encoding HEK293T cells were washed prior to reading the Ca^2+^ flux, we quantified the cell loss that may occur when media is aspirated from a 384-well and replaced. Taking advantage of the double injection feature of the FlexStation II 384, we injected 10 µL of 0.1% Triton X-100 (final conc.) after the cells were stimulated by their respective ligands. This lyses the cells, which release GCaMP6s, which cause a measurable increase in GFP fluorescence as they come into contact with free extracellular Ca^2+^. In this way, the corrected mean RFU from the second injection quantifies the total cell count in the non-washed (striped) or washed (solid) wells. The wash step reduces the fluorescence, indicating about a 9% cell loss. Data are mean ± SEM from three independent experiments with 66 technical replicates each.

| Name | Percentile Rank | Average FPKM* | SD FPKM* |
| --- | --- | --- | --- |
| **Adenosine Receptors** | | | |
| ADORA2B | 86.999 | 10.859 | 0.676 |
| ADORA2A | 80.626 | 4.185 | 1.002 |
| ADORA1 | 73.911 | 1.153 | 0.161 |
| ADORA3 | 50.888 | 0.015 | 0.025 |
| **P2Y Receptors** | | | |
| P2RY11 | 90.671 | 17.846 | 0.725 |
| P2RY1 | 83.465 | 6.529 | 0.768 |
| P2RY2 | 70.060 | 0.539 | 0.151 |
| P2RY4 | 60.484 | 0.092 | 0.047 |
| P2RY6 | 52.043 | 0.019 | 0.033 |
| P2RY12 | 50.134 | 0.012 | 0.010 |
| P2RY14 | 48.305 | 0.006 | 0.011 |
| P2RY8 | 47.624 | 0.004 | 0.007 |
| P2RY10 |  | 0.000 | 0.000 |
| P2RY13 |  | 0.000 | 0.000 |

^*^  = Fragments Per Kilobase of transcript per Million mapped reads

**Table S1 Adenosine receptors and P2Y receptors ranked in order of expression in HEK293T cells**

Data were obtained from RNASeq analysis of untransfected HEK293T cells and the receptors are ranked in order of percentile rank of all expressed genes ([Barbash et al., 2019](#_ENREF_1)). Receptors with a Percentile Rank between 0 and 46% have no detectable expression in HEK293T cells.

**References**

Barbash, S., Persson, T., Lorenzen, E., Kazmi, M.A., Huber, T., and Sakmar, T.P. (2019). Detection of Concordance between Transcriptional Levels of GPCRs and Receptor-Activity-Modifying Proteins. iScience *11*, 366-374.
